# Comparative transcriptional profiling-based identification of raphanusanin-inducible genes

**DOI:** 10.1186/1471-2229-10-111

**Published:** 2010-06-16

**Authors:** Moehninsi, Kenji Miura, Haruyuki Nakajyo, Kosumi Yamada, Koji Hasegawa, Hideyuki Shigemori

**Affiliations:** 1 Graduate School of Life and Environmental Sciences, University of Tsukuba, Ibaraki 305-8572, Japan; 2 KNC Laboratories Co, Ltd, Hyogo 651-2271, Japan

## Abstract

**Background:**

Raphanusanin (Ra) is a light-induced growth inhibitor involved in the inhibition of hypocotyl growth in response to unilateral blue-light illumination in radish seedlings. Knowledge of the roles of Ra still remains elusive. To understand the roles of Ra and its functional coupling to light signalling, we constructed the Ra-induced gene library using the Suppression Subtractive Hybridisation (SSH) technique and present a comparative investigation of gene regulation in radish seedlings in response to short-term Ra and blue-light exposure.

**Results:**

The predicted gene ontology (GO) term revealed that 55% of the clones in the Ra-induced gene library were associated with genes involved in common defence mechanisms, including thirty four genes homologous to *Arabidopsis *genes implicated in R-gene-triggered resistance in the programmed cell death (PCD) pathway. Overall, the library was enriched with transporters, hydrolases, protein kinases, and signal transducers. The transcriptome analysis revealed that, among the fifty genes from various functional categories selected from 88 independent genes of the Ra-induced library, 44 genes were up-regulated and 4 were down-regulated. The comparative analysis showed that, among the transcriptional profiles of 33 highly Ra-inducible genes, 25 ESTs were commonly regulated by different intensities and duration of blue-light irradiation. The transcriptional profiles, coupled with the transcriptional regulation of early blue light, have provided the functional roles of many genes expected to be involved in the light-mediated defence mechanism.

**Conclusions:**

This study is the first comprehensive survey of transcriptional regulation in response to Ra. The results described herein suggest a link between Ra and cellular defence and light signalling, and thereby contribute to further our understanding of how Ra is involved in light-mediated mechanisms of plant defence.

## Background

**Figure 1:**
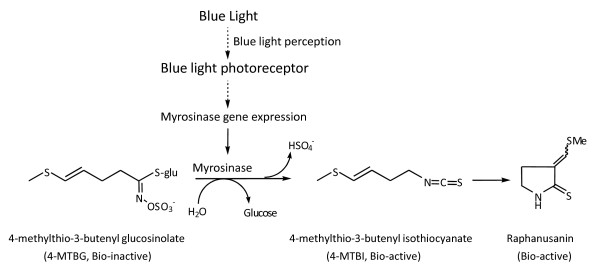
**Formation of raphanusanin growth inhibitor by hydrolysis of 4-MTBG by myrosinase**. Phototropic stimulation promotes myrosinase activity on the illuminated side of radish hypocotyls, releasing bioactive, 4-MTGI from inactive 4-MTBG and simultaneously producing bioactive raphanusanin (modified from Yamada et al., 2003).

Raphanusanin (3-methylthio-methylene-2-pyrrolidonethione) (Ra) can be isolated from radish seedlings grown under illumination and plays a role in the light-induced inhibition of hypocotyl growth [1]. When applied unilaterally, Ra suppresses hypocotyl growth on the treated side more than on the opposite side, inducing a differential growth gradient that causes the hypocotyl to bend towards the side of application [2,3]. Blue-light irradiation rapidly decreases the 4-methylthio-3-butenyl glucosinolate (MTBG) content and abruptly increases the content of 4-methylthio-3-butenyl isothiocyanate (MTBI) and raphanusanin in the radish hypocotyls within 30 min after the onset of irradiation [4]. When MTBG, MTBI, and raphanusanin at endogenous levels were applied unilaterally to etiolated hypocotyls, MTBI and raphanusanin caused hypocotyls to bend, but MTBG induced no activity. Blue-light irradiation promoted myrosinase (thioglucosidase) activity, which releases MTBI from MTBG, in hypocotyls after 10 min, although the enzyme activity in the dark controls did not change [4]. Some chemical studies on 4-methylthio-3-butenyl isothiocyanate (MTB-ITC) have been carried out, where MTB-ITC has been spontaneously converted into raphanusanins in MeOH-H_2_O or H_2_O solution [5]. The biosynthetic pathway of Ra is shown in Figure 1. Phototropic stimulation promotes myrosinase activity on the illuminated side of radish hypocotyls, releasing bio-active 4-methylthio-3-butenyl isothiocyanate (4-MTBI) from bio-inactive 4-methylthio-3-butenyl glucosinolate (4-MTBG), and simultaneously producing bio-active Ra. Sakoda et al. demonstrated that the IAA-mediated transverse microtubule reorientation is significantly inhibited by the simultaneous addition of Ra analogues [6]. Moreover, Nakajima et al. showed that Ra inhibited apical dominance in pea seedlings [7]. There are certainly additional ways in which Ra may help a plant to adapt to the prevailing light environments, and these may be discovered through further photophysiological, cellular, biochemical, and genetic testing. We demonstrated the physiological role of Ra in differential growth and identified the first four genes shown to be induced by Ra using modified Differential Display RT-PCR (DD-RT-PCR). We characterised one of these genes, *RsCSN3*, as an essential element in the inhibition of hypocotyl growth [3]. However, our first attempt using the DD-RT-PCR method was limited to the identification of a large number of genes induced by Ra. The comprehensive understanding of the functional activity of Ra still remains elusive. To search for additional components required for the understanding of the roles of Ra, we constructed the Ra-induced gene library using the Suppression Subtractive Hybridisation (SSH) technique. The use of a rapid and sensitive mRNA expression comparison technique (SSH) and its application in comparative studies with light will be crucial to revealing the possible roles of Ra and its functional coupling to light signalling. Here, we present a comparative investigation of gene regulation in radish seedlings in response to short-term Ra and blue-light exposure. Among the transcripts identified to be up-regulated in response to raphanusanin, we observed many genes that are related to the multiple signalling of cellular defence. This observation has allowed us to infer raphanusanin regulatory roles for a large fraction of products associated with movement, transporters, protein metabolism, protein kinases, and hydrolases.

## Results

### Raphanusanin-induced genes that are linked to cellular defence

**Figure 2:**
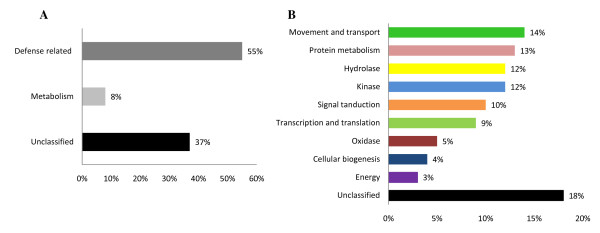
**Functional classification of raphanusanin-induced cDNA clones**. A total of 88 genes were classified in to their physiological functions, A, and biochemical functions, B, based on the BLASTX homology search.

To obtain Ra-induced genes, we subtracted the cDNAs in the Ra-untreated four-day-old radish seedlings from those in the samples treated with Ra (approximate endogenous level, 50 ng) 15 min (see additional file 1). Starting with two micrograms of poly-A^+ ^RNA prepared from Ra-treated samples, 287 cDNA clones induced by Ra were obtained (Table 1). The nucleotide sequences of all of the clones were determined by sequencing the inserts from both ends, altogether generating 574 ESTs (287 × 2 = 574; Table 1). The Ra-induced cDNA library digested with *Rsa*I contained on average 400 bp inserts. Thus, the sequences from both ends were overlapping. All of the sequences were then assembled by the Phrap program (Codon Code Aligner Sequence Assembler v3.0.1) and classified into 101 non-redundant forms after building the contigs (Table 1). All of the 101 non-redundant sequences were submitted to the DDBJ database with accession numbers assigned (see additional file 2). The sequence of each non-redundant EST was identified by similarity search in the NCBI non-redundant public sequence database (nr) [8]. If a gene contained the *Rsa*I digestion site, two cDNA clones could be identified. Therefore, neighbouring clones were joined by alignment with subject sequences obtained by a BLAST search. After the BLAST search, 88 Ra-induced independent genes were identified, and 13 were duplicates. Of the 88 independent genes, 77 were demonstrated to have significant homology with *Arabidopsis thaliana *genes, 9 were homologous with other organisms such as *Brassica *and chicken, and 2 were novel ESTs (Table 1). The 88 independent genes were classified into three physiological associations or ten biochemical functional categories based on the best BLASTX match of the corresponding ESTs against NCBI non-redundant protein database (expect value < 0.01) or TAIR Arabidopsis protein database (Figure 2 and see additional file 2).

**Table 1:** Summary of the structural analysis of the raphanusanin-induced cDNA library

Category	Number of sequences
**Redundancy consideration**	
Clones	287
Sequenced	574
Non-redundant sequence	101
Independent gene	88
**Ra-induced genes with high similarity against BLASTX**	
*Arabidopsis thaliana *EST	77
*Raphanus sativus *EST	3
*Brassica napus *EST	4
*Brassica oleracea *EST	1
*Gallus gallus *EST	1
Novel EST	2

Forty-eight of the 88 Ra-inducible genes (55%) were categorised as related to cellular defence, and 7 genes (8%) were categorised as related to energy metabolism (Figure 2A). On the other hand, 33 of the 88 Ra-inducible genes (37%) could not be assigned to any physiological category (Figure 2A and see additional file 2). A high portion of these genes are involved in common defence mechanisms, including CIPK1, PTI1, GTF, RPK (ERECTA), HSP90, MBP1, CAMTA3, ACC oxidase (ACCO), GTPase, UBQ, DRP, PLC, and PLD [9-18]. Furthermore, nine genes function in the response to abiotic stress, including USP, SDR, RALF 23, and GASA4 [19-22]. There were also five genes known to be involved in toxic catabolic processes and in the response to oxidative stress, such as catalase2 (CAT2), peroxidase, NTRA, and MetE [23-25]. In addition, two genes, pescadillo and coproporphyrinogen III oxidase (CPOX), are induced by DNA damage and protein lesions [26,27].

Based on biochemical classification (Figure 2B and see additional file 3), 31 genes among the 88 Ra-inducible genes were categorised as signalling-related, such as kinases (10 genes, 12%), protein metabolism/binding (11 genes, 13%), and signal transduction (10 genes, 12%). The leucine-rich repeat (LRR) disease-resistance protein kinases, serine/threonine, and tyrosine protein kinases, such as CIPK1, PKC, MAK, and Pti1, were included in the kinase category. Six genes involved in the ubiquitin-proteasome pathway, such as CSN3, KEG, CUL1, UMP1, UBQ3, and RING finger [11,28-32], were categorised into protein metabolism. In addition, the nine genes involved in signal transduction are all known to be associated with defence against environmental stress (e.g., PLC, PLD, DRP, and ERECTA). Other than the genes involved in housekeeping functions, those associated with signal transduction, kinases, movement and transporter, and hydrolase were the most abundant (Figure 2B and see additional file 3). These results indicate that Ra induces changes in the genetic network in preparation for a distinct phase of cellular communication. In addition, a bioinformatic analysis revealed that 34 genes among the 88 Ra-induced genes homologous to *Arabidopsis *genes were implicated in the function of the R-gene-triggered resistance in programmed cell death (PCD), as shown in Table 2.

**Table 2:** Annotation of Ra-induced genes homologous to *Arabidopsis *genes that are involved in R-gene-triggered resistance in PCD pathway

Gene family	No. of gene	Putative function	Reference
**Protein kinase**		R proteins as regulatory adaptors in plant apotosomes	
Leucine Rich Repeat transmembrane protein	**4**		Torres et al. (2006); Hofius et al. (2007) [56,47]
Serine-theorine protein kinases (CIPK1, Pit1, Mak)	**3**		Schwachtje et al. (2006); Hofius et al. (2007) [16,47]
Receptor-like kinase (ERECTA)	**1**		Chen et al. (2003) [49]
Glycoprotein	**1**		Wycoff et al. (1995) [48]

**Oxidative & electrophilic stress**	**3**	Signal activators in tranduction network of HR-related PCD	Zhang and Kirkham (1994); Arner and Holmgren (2000); Laloi et al. (2001); Heinemann et al. (2008); Zhu et al. (2008) [60,57,58,34,66]
NTRA,CAT2, Peroxidase			
**Channel activators**	**4**		Gelli et al. (1997); Nurnberger et al. (2004); Zimmermann et al. (1997); Galon et al. (2008) [52,51,53,37]
CAMTA3, KT, POT, H^+ ^ATPase			
**Lignin-associated genes**	**3**		
DRP, Peroxidase , RALF23			Franssen and Bisseling (2001); Pearce et al. (2001); Navarro et al. (2004) [19,84,35]
**GTPases**	**2**		
(Rab-like small GTPase, FfG SRP GTPase)			Ono et al. (2001); Cheung et al. (2008) [110,111]
**Chloroplast target gene**	**1**		
CPOX			Ishigawa (2005) [27]

**Ubiquitin-proteosome system**	**7**	Modulators of R-gene-triggered resistance	Zou et al. (2006);Liu et al. (2002); Shirasu et al. (2003); Ren et al. (2005); Stone et al. (2006); Kawasaki et al. (2005); Taupp et al. (2008). [9,28,67,71,30,70,32]
HSP90, CSN3, CUL1, KEG, C3HC4-type Ring Finger, UBQ3, PMF1			

**Stress responsive hormones**	**3**	R-gene function in hormonal control of PCD activation	Ohtsubo et al. (1999); Ciardi et al. (2001); Desikan et al. (2001); Kim et al. (2003); Hudgins et al. (2004) [73,74,77,38,12]
ACC oxidase, MBP1, CAMTA3			

**Phosphatidic acid precursors**	**2**	Modulators of lipid-based signals in PCD	Smith et al. (2004); Chen et al., 2007; Ramina et al. (2007) [42,49,18]
PLC, PLD			

### The determination of reference genes for qRT-PCR

In order to evaluate transcriptional regulation, the selection of the most appropriate and stable housekeeping gene as a control is necessary. The information for choosing the proper housekeeping gene in the radish system is still limited. Therefore, the transcript levels of seven housekeeping genes commonly used in plant gene expression analysis, actin8, ubiquitin3, 18S rRNA, tubulin α-6, rRNA protein (L4), initiation factor 2 (eIf2), and elongation factor 1B-alpha (ef1α), were measured before and after the Ra treatment (see additional file 4). The entire experiment was performed in triplicate, and the results were combined for statistical analysis. The cycle threshold (C_T_) of each transcript was compared. Tubulin α-6 was the most abundant (lowest C_T_) transcript and L4 was the least abundant (see additional file 4 and additional file 5). The ANOVA F-test of differences among time points after Ra treatment indicated that the transcript levels of three genes, ef1α, eIf2, and L4, were not significantly altered before and after Ra treatment in radish seedlings. Two of these three genes, ef1α and eIf2, had a small coefficient of variation (CV) (see additional file 5). Both consistency across time points (low slope) and high predictability (low CV) are desired for a control. The stability index was calculated based on the product of the slope and the CV (see additional file 5). The gene with the lowest stability index provides the best control. In this study, ef1α had the lowest stability index and eIf2 had the second lowest as evidenced by their low slope and CV. Therefore, ef1α and eIf2 were used to normalise the expression levels of the genes of interest.

### Defence-related genes are positively regulated in response to raphanusanin

**Figure 3:**
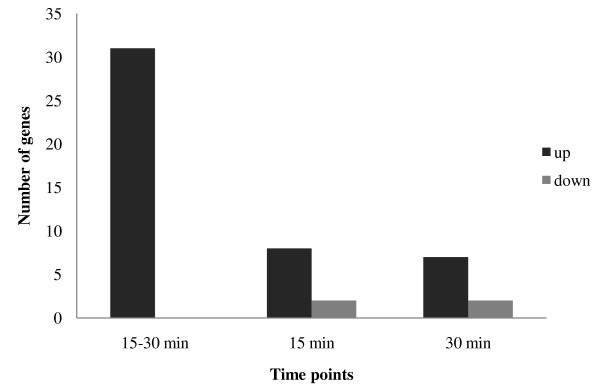
**Genes differentially regulated in a time dependent manner in 4-d-old etiolated seedlings subjected to 50 ng of raphanusanin (Ra) treatment over time**. Number of genes up-and down-regulated by Ra after 15 min and/or 30 min time points. To clarify, the genes expression levels (≤ 1.5 or ≥ 0.6) were excluded from the total number of differentially regulated genes.

Fifty expressed sequence tags (ESTs) from various functional categories were selected from among the 88 independent genes from the Ra-induced library to confirm the induction of transcripts by quantitative RT-PCR. Three independent preparations of mRNA for each biological replicate were pooled to eliminate inconsistencies due to sampling. Three independent experiments were carried out from three independent pools. An analysis of the genes exhibiting changes in expression greater than 1.5-fold or less than 0.6-fold at both time points (15 min and/or 30 min) in comparison to the untreated samples revealed that 44 genes were up-regulated and 4 were down-regulated (Table 3 and Figure 3). Some genes were highly expressed during the first 15 min, while others were more pronounced at 30 min. An analysis of genes with assigned functional categories revealed that several metabolic processes linked to common protective functions, including the biosynthesis of stress-activated hormones, reactive oxygen scavenging enzymes, signal transducers, and components of the protein degradation system, were up-regulated (Table 3). The number of up-regulated genes and the expression levels were substantially greater at 15 min than at 30 min **(**Figure 3). This difference could be attributed to the early activation of genes underlying the mechanistic response to Ra. Among the down-regulated genes, the transcript levels of RMB1 and RMB2 were lower at 15 min, whereas those of CND41 and PKC were lower at 30 min. The highly expressed genes, NTRA (>70) and peroxidase (>5), are reactive oxygen-scavenging enzymes involved in the removal of superoxide radicals [33,34]. In addition, DRP, ERECTA (>10), and CAMTA 3 (>3) are signal transducers that rescue cells from pathogenic attacks [35-37]. Two genes, ACCO and MBP1 (>10), encode the genes involved in induction of the major defence-related hormones, ethylene and jasmonate acid, respectively [38,39]. It should be noted that seven genes (UBQ3, PMF1, CUL1, CSN3, KEG, and C_3_HC_4_-type Ring Finger, HSP90) involved in the proteolytic pathway were up-regulated in response to Ra. These genes are implicated in the regulation of a vast array of biological processes, including the cell cycle [40], apoptosis [41], the adaptive immune system [42], plant growth regulation [43,44], and responses to oxidative stress [45,46]. The most highly expressed genes were defence-associated genes, implicating the correlation of Ra with cellular defence.

**Table 3:** Annotation of up- and down-regulated genes in the Ra^+^-Ra^- ^library

**Family**^**a**^	Genome initiative No. and Putative function	Expression ratio		**Function **^**b**^
		15 min	30 min	
NTRA	AT2G17420 NTRA (NADPH-dependent thioredoxin reductase 2)	74.3 ± 11.2*	**4.18 ± 1.9**	1
DRP	AT4G23690 Disease resistence response protein (DRP)	33.7 ± 6.4*	9.65 ± 2*	2
ERECTA	ATG26330 Receptor Protein kinase ERECTA (ER)	10.88 ± 2.3*	15.82 ± 1*	2
CCR4-NOT	AT3G44260 CCR4-NOT transcription complex protein	4.86 ± 0.4*	3.4 ± 0.2*	3
CSN3	AB-355980 cop9 signalosome subunit 3	4.2 ± 0.7*	**1.5 ± 0.3**	4
Kinesin	AT2G21380 kinesin motor-protein-related	3.93 ± 0.8*	**3.97 ± 1**	5
UMP1	AT1G67250 Proteasome maturation factor UMP1	3.9 ± 0.6*	3 ± 0.4*	4
ACCO	X81628.1 ACC oxidase	3.67 ± 0.2*	**3.96 ± 1.4**	1
USP	AT3G53990 Universal stress protein (USP) family protein	1.93 ± 0.2*	**2.02 ± 0.6**	2
MBP 1	Y11482 Myrosinase binding protein MBP1	**3.6 ± 0.5**	5.1 ± 0.8*	4
KT	AT4G19960 potassium iron transporter (KT)	**3.4 ± 0.9**	2.24 ± 0.4*	5
CAMTA 3	AT2G22300 Calmodulin-binding transcription activator 3 (CAMTA 3)	**1.65 ± 0.2**	2.03 ± 0.1*	3
PTI1	AT2G30740 PTI1-like protein tyrosine kinase	2.18 ± 0.2*	**3.12 ± 0.6**	6
CUL1	AT4G02570 Cullin-like protein, a subunit of E3 ubiquitin ligase	**2.83 ± 0.4**	**1.86 ± 0.2**	4
SDR	AT4G09750 short-chain dehydrogenase	**2.37 ± 0.3**	**2.2 ± 0.0.4**	7
Peroxidase	AT3G32980 Heme-dependent peroxidase	5.5 ± 0.5*	3.43 ± 0.3*	1
HSP90	AT4G24190 SHD (SHEPHERD) HSP90	1.38 **± **0.3	1.26 **± **0.1	4
KEG	AT5G13530 RING E3 ligase protein (KEG)	2.13 ± 0.1*	0.8 **± **0.1	4
CIPKI	AT2G30360 SNF 1-related protein kinase	**2.19 ± 0.4**	2.9 ± 0.38*	6
GH3	AT5G20950 Glycosyl hydrolase family 3 protein(GH3)	**2.1 ± 0.3**	1.4 ± 0.1	7
CESA5	AT5G09870 Cellulose synthase 5- transferase	**1.5 ± 0.1**	1.95 ± 0.1*	5
CAT2	AF139538 Catalase2	1.97 ± 0.2*	**1.6 ± 0.3**	1
Glycoprotein	AT1G14710 Hydroxy proline rich glycoprotein family	1.88 ± 0.2*	0.9 ± 0.1	10
POT	AT3G16180 Proton-dependent oligopeptide transport (POT) family protein	1.82 ± 0.1*	1.05 ± 0.1	5
LRT	AT2G31880 Leucine-rich repeat tranmemberane protein kinase	**1.91 ± 0.5**	**1.7 ± 0.2**	6
Clathrin	AT1G60780 Clathrin adaptor complexs medium subunit family protein	1.8 ± 0.14*	1.2 ± 0.1	5
PKC	AY835401.1 Protein Kinase C conserved region 2	**1.7 ± 0.2**	0.38 ± 0.1*	6
Pescadillo	AT5G14520 Pescadillo-related protein	**1.7 ± 0.2**	**2.12 ± 0.4**	3
3KCS4	AT1G19440 Very long-chain fatty acid condensing enzyme	**1.6 ± 0.1**	**1.5 ± 0.2**	5
Profilin	AT2G19760 Profilin1	**1.59 ± 0.1**	2.23 ± 0.1*	9
Dehydrin	AT2G39750 Dehydration-responsive family protein	**1.67 ± 0.2**	**1.8 ± 0.3**	10
PGβ1	AT1G70370 Polygalacturonase isoenzyme 1 beta subunit homolog	**1.56 ± 0.2**	1.44 ± 0.2	10
MAK	AT5G45430 serine/threonine-protein kinase Mak	**1.6 ± 0.3**	1.25 ± 0.1	6
3PGDH	AT1G17745 3-phosphoglycerate dehydrogenase	1.65 ± 0.1*	**1.8 ± 0.2**	7
Ring Finger	AT3G09760 C3HC4-type Ring Finger	**1.55 ± 0.2**	1.92 ± 0.1*	4
G6PDH	AT5G35790 Glucose 6 -phosphate dehydrogenase	0.69 ± 0.1	**1.6 ± 0.2**	7
GTPase	AT5G53570 Gtpase activator protein for Rab-like GTPase-like protein	**1.5 ± 0.1**	**2 ± 0.2**	2
UBQ3	At5g03240 UBQ3	1.4 **± **0.1	2.35 ± 0.2*	4
CND41	AT3G18490 aspartyl protease family protein	1.3 ± 0.1	0.01 ± 0.1*	4
MetE	AT5G17920 Cobalamin-independent methionine synthase	1.4 ± 0.1	1.13 ± 0.1	4
RALF	AT3G16570 Rapid Alkalization Factor 23 (RALF23)	1.31 ± 0.2	**2.3 ± 0.4**	2
PPEase	AT2G26870 Phosphoesterase family protein	1.3 ± 0.1	1.3 ± 0.1	7
CPOX	AF375424 Coproporphyrinogen III oxidase	1.3 ± 0.1	1.45 ± 0.1	1
BURP	AT1G70370 Polygalacturonase isoenzyme 1 beta subunit homolog	1.3 ± 0.1	1.1 ± 0.1	10
GASA4	AT5G15230 GASA4 (GAST1 protein homolog)	1.27 ± 0.2	**3.1 ± 0.7**	10
PLC	AAD26119.1 Phosphoinositide- specific phospholipase C	1.23 ± 0.1	**1.78 ± 0.2**	2
MAG2	AT3G47700 MAG2(chromosome structure maintenance protein-related)	1.2 ± 0.1	1.7 ± 0.1*	5
GTF	AT1G19710 Glycosyl transferase family 1 protein	1.07 ± 0.1	1.95 ± 0.1*	5
RMB2	AB042187 Myrosinase (RMB2)	**0.45 ± 0.1**	1.66 ± 0.1*	7
RMB1	AB04218 Myrosinase (RMB1)	**0.52 ± 0.1**	**1.52 ± 0.2**	7

^a ^one representative clone for each gene is shown. Three independent biological replicates were performed. Value **± **s.e. indicates expression ratio of Treatment/Control after normalization **± **standard error of the mean (n = 3).Genes in the table are listed in decreasing expression ratios according to genes in the Ra^+^-Ra^- ^library. Genes that differ significantly (student t test p-value less than 0.05) in their expression between the treatment and control are marked with an asterisk (*). Bold represents the expression ratios (≥ 1.5 or ≤ 0.6). ^b^The functional categories are, 1: Oxidase, 2: signal tranduction, 3: transcription and translation, 4: protein metabolism/binding, 5: movement and transport, 6: kinases, 7: Hydrolases, 8: Energy, 9: Cellular biogenesis, 10: Unclassified. The values shown represent fold change (treatment vs. control).

### Raphanusanin is functionally coupled with the early blue-light response

**Figure 4:**
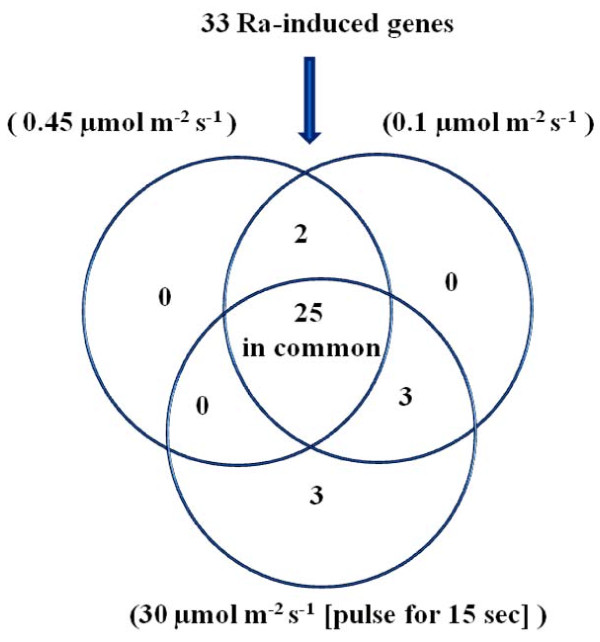
**Response of raphanusanin (Ra)-induced genes to three different blue light (BL) intensities (0.45 μmol m**^**-2 **^**s**^**-1**^**), BL (0.1 μmol m**^**-2 **^**s**^**-1**^**), BL (30 μmol m**^**-2 **^**s**^**-1**^**, pulse for 15 sec)**. Venn diagram indicates the differential expression of genes upon the three different treatments of BL at different time points.

**Figure 5:**
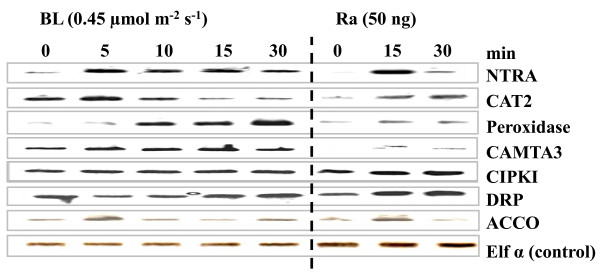
**BL and Ra induce the genes related to growth inhibition and cellular defence**. Time courses of transcript levels of seven genes involved in growth inhibition pathways relative to controls following treatment with BL and Ra at the indicated concentrations were validated by reverse transcriptase-polymerase chain reaction (RT-PCR). Transcript levels of seven genes, NADPH-dependent thioredoxin reductase A (NTRA), catalase2 (CAT2), peroxidase, calmodulin- binding transcription factor 3 (CAMTA3), SNF-1 related protein kinase (CIPK1), disease resistance protein (DRP), and ACC oxidase. The bottom panels show the elongation factor α (ef1α) as loading control. Primers for all genes are specific to transcripts from the respective cDNA. The data represents the typical gel image of one of three replicates. BL: blue light; Ra: raphanusanin.

Because Ra is a blue light (BL)-induced growth inhibitor of radish seedlings [3], elucidating the relationship between Ra and blue light is a crucial step in understanding the proper roles of Ra. To obtain information regarding the strict specificity of the Ra involvement in the early light-mediated development, four-day-old etiolated radish seedlings were irradiated with three different kinds of BL fluences. The transcriptional profiles of 33 highly Ra-inducible genes were analysed in response to BL. The 25 ESTs were commonly regulated by the different intensities and durations of BL irradiation (Figure 4). Many stress-related genes (e.g., CUL1, CSN3, and MBP1) were observed to respond early during the first five minutes of high-fluence pulses, while they increased dramatically over time in the continuous light of low fluence, potentially resulting from the reciprocity of the dose response (Table 4). Two genes (PKC and SDR) were specific to the continuous-light response, and three genes (PLC, USP, and 3KCS4-21) were specific to the pulsed-light response. There were also a few genes regulated by Ra and BL in the opposite manners (e.g., RMB2, and CAT). The observed responses for different fluences reveal that the range of fluences over which the response specificities to continuous or pulsed light may be attributed to the action of phot1 at lower fluence rates (0.1-50 μmol m^2 ^s^-1^) and phot2 at higher fluence rates (1-250 μmol m^2 ^s^-1^). Several genes involved in growth inhibition were up-regulated by Ra and BL (Figure 4 and Table 4). To validate, the expression levels of seven genes participating in growth inhibition were measured by semi-quantitative RT-PCR analysis (Figure 5). These results suggest that blue light facilitates the expression of Ra-inducible genes.

**Table 4:** Summary of expression ratio of 33 ESTs from highly regulated Ra-induced genes over BL at different fluences at indicated time points versus dark-grown seedlings

**Family**^**a**^	Function	5 min			15 min			30 min		
		**Continuous1**	**Continuous2**	**pulse**	**Continuous1**	**Continuous2**	**pulse**	**Continuous1**	**Continuous2**	**pulse**

Ubi-3	1	**2.8 ± 0.6**	1.34 ± 0.2	**2.5 ± 0.5**	6 ± 0.7*	2.7 ± 0.2*	2.8 ± 0.4*	4.3 ± 0.6*	**1.8 ± 0.5**	**2.3 ± 0.3**
CSN3	1	1.5 ± 0.1	1.23 ± 0.1	3.8 ± 0.5*	2.9 ± 0.3*	1.97 ± 0.2*	1.13 ± 0.1	2.02 ± 0.2*	1.3 ± 0.1	0.98 ± 0.1
Zinc Finger	1	1.3 ± 0.2	0.9 ± 0.2	2.6 ± 0.3*	**2.68 ± 0.6**	**1.5 ± 0.6**	1.23 ± 0.2	**1.95 ± 0.4**	**2.2 ± 0.3**	0.93 ± 0.3
PMF1	1	1.5 ± 0.2	**3.2 ± 0.5**	**2.14 ± 0.4**	**2 ± 0.2**	2.8 ± 0.3*	1.2 ± 0.2	2.5 ± 0.2*	**3.8 ± 1**	1.12 ± 0.1
CND41	1	0.8 ± 0.2	0.78 ± 0.1	1.04 ± 0.1	0.62 ± 0.1	**0.25 ± 0.2**	1.96 ± 0.2*	**1.53 ± 0.3**	1.25 ± 0.2	**1.81 ± 0.3**
CUL1	1	1.19 ± 0.1	**1.65 ± 0.1**	4.1 ± 0.7*	**1.56 ± 0.2**	1.85 ± 0.1*	1.49 ± 0.1	2.7 ± 0.2*	**2.8 ± 0.6**	0.89 ± 0.2
NTRA	2	**2.7 ± 0.4**	**1.8 ± 0.5**	**2.3 ± 0.3**	**3.2 ± 0.6**	**2.98 ± 0.6**	**2.15 ± 0.4**	1.3 ± 0.1	1.14 ± 0.2	0.92 ± 0.1
ERECTA	2	5.3 ± 0.6*	16.5 ± 2.3*	**1.8 ± 0.2**	9.16 ± 1.7*	**5.5 ± 1.5**	**2.2 ± 0.3**	3.23 ± 0.3*	**3.85 ± 0.8**	**1.9 ± 0.2**
CAT	2	1.37 ± 0.2	0.89 ± 0.1	0.7 ± 0.1	0.42 ± 0.1*	**0.22 **± 0.1*	0.58 ± 0.1	0.35 ± 0.1*	1 ± 0.2	2.2 ± 0.2*
MBP1	2	1.01 ± 0.1	0.88 ± 0.1	**2.1 ± 0.3**	2.76 ± 0.4*	**1.86 ± 0.3**	1.12 ± 0.1	**4.32 **± 1	**2.08 ± 0.5**	1.3 ± 0.4
CIPKI	2	**1.8 ± 0.3**	0.89 ± 0.2	1.05 ± 0.1	2.39 ± 0.2*	1.34 ± 0.2	1.4 ± 0.2	2.98 ± 0.4*	**2 ± 0.4**	**2.2 ± 0.6**
CAMTA3	2	0.77 ± 0.1	1.21 ± 0.1	0.98 ± 0.1	1.98 ± 0.2*	1.8 ± 0.1*	2.63 ± 0.2*	1.03 ± 0.1	1.3 ± 0.1	1.2 ± 0.3
DRP	2	**2.03 ± 0.4**	**4.9 ± 1.2**	**3.8 ± 0.6**	2.2 ± 0.2*	3.8 ± 0.5*	**2 ± 0.3**	**3.9 ± 0.9**	5.98 ± 0.2*	**1.79 ± 0.3**
RMB2	2	1.22 ± 0.1	1.35 ± 0.3	0.91 ± 0.1	**1.89 ± 0.3**	**1.81 ± 0.2**	1.2 ± 0.1	1.43 ± 0.2	1.25 ± 0.1	1.01 ± 0.2
Glycoprotein	2	0.6 ± 0.1	0.56 ± 0.2	0.78 ± 0.1	**1.73 ± 0.2**	**2.57 ± 0.4**	0.75 ± 0.1	3.22 ± 0.5*	**2 ± 0.8**	2.4 ± 0.3*
ACCO	2	1.12 ± 0.2	1.09 ± 0.1	3.14 ± 0.3*	**1.98 ± 0.2**	**1.83 ± 0.3**	**1.56 ± 0.3**	**3.15 ± 0.7**	**1.93 ± 0.5**	0.91 ± 0.1
LRT	2	1.06 ± 0.1	1.12 ± 0.1	0.9 ± 0.1	0.95 ± 0.2	0.85 ± 0.2	0.8 ± 0.1	**4.3 ± 1.9**	2.97 ± 0.4*	**9.5 ± 3**
Kinesin	3	**1.9 ± 0.5**	1.45 ± 0.4	**2.55 ± 0.8**	3.2 ± 0.2*	**2.65 ± 0.7**	**3.1 **± 1.1	**4.33 ± 0.9**	2.15 ± 0.2*	1.41 ± 0.3
CCR4-NOT	4	2.89 ± 0.3*	3.67 ± 0.2*	2.3 ± 0.2*	**4.5 ± 1.2**	**1.88 ± 0.3**	1.3 ± 0.1	6.1 ± 1.2*	**1.98 ± 0.5**	**1.68 ± 0.2**
PPFP	4	1.48 ± 0.1	**6.8 ± 1.5**	**2 ± 0.2**	**1.8 ± 0.2**	3 ± 0.4*	0.33 ± 0.1*	1.39 ± 0.2	**2.67 ± 0.6**	**1.6 ± 0.5**
PPEase	4	**2.4 ± 0.3**	1.2 ± 0.2	7.9 ± 1.3*	2.3 ± 0.2*	**2.4 ± 0.4**	4.5 ± 0.7*	3.3 ± 0.3*	2.8 ± 0.2*	1.25 ± 0.2
PTI1	4	**2.8 ± 0.5**	4 ± 0.6*	**2.05 ± 0.6**	**2 ± 0.3**	**1.5 ± 0.5**	0.98 ± 0.2	**1.7 ± 0.1**	1.1 ± 0.1	**1.98 ± 0.3**
KT	4	0.73 ± 0.1	1.35 ± 0.2	**1.8 ± 0.2**	**1.65 ± 0.1**	**1.5 ± 0.6**	0.38 ± 0.1*	**1.55 ± 0.3**	1.35 ± 0.2	0.82 ± 0.1
SDR	3	0.85 ± 0.2	0.7 ± 0.1	1.09 ± 0.1	2.64 ± 0.1*	**1.8 ± 0.2**	0.9 ± 0.1	1.09 ± 0.1	1.23 ± 0.1	0.85 ± 0.1
PKC	4	**0.5 ± 0.3**	0.25 ± 0.2*	1.4 ± 0.1	0.77 ± 0.1	0.18 ± 0.1*	0.78 ± 0.2	0.65 ± 0.1	0.83 ± 0.1	1.3 ± 0.1
Pescadillo	2	0.5 ± 0.1*	**0.6 ± 0.2**	0.8 ± 0.2	0.82 ± 0.1	0.23 ± 0.1*	3.37 ± 0.4*	1.25 ± 0.5	0.8 ± 0.1	**2.35 ± 0.3**
GTPase	2	0.94 ± 0.1	0.83 ± 0.1	2.6 ± 0.3*	1.13 ± 0.1	**0.42 ± 0.2**	**3.55 ± 0.7**	0.92 ± 0.1	**0.47 ± 0.3**	3.1 ± 0.3*
GH3	3	1.1 ± 0.1	1.17 ± 0.1	1.4 ± 0.3	1.34 ± 0.2	**1.8 ± 0.3**	**2.1 ± 0.3**	1.09 ± 0.1	1.12 ± 0.1	**2.3 ± 0.5**
PLC	4	0.98 ± 0.1	0.87 ± 0.2	1.32 ± 0.1	0.7 ± 0.1	1.12 ± 0.1	0.93 ± 0.1	1.1 ± 0.3	0.7 ± 0.1	2.89 ± 0.4*
PGβ1	4	0.98 ± 0.2	1.16 ± 0.1	**1.55 ± 0.2**	1.3 ± 0.1	**1.85 ± 0.4**	3.3 ± 0.5*	1.4 ± 0.1	**1.55 ± 0.2**	**2.5 ± 0.5**
Peroxidase	2	2.76 ± 0.3*	0.95 ± 0.1	**2.5 ± 0.6**	**1.8 ± 0.4**	2.5 ± 0.2*	1.2 ± 0.1	1.23 ± 0.1	1.05 ± 0.1	1.3 ± 0.3
USP	2	1.03 ± 0.2	0.9 ± 0.2	0.69 ± 0.1	1 ± 0.1	1.2 ± 0.1	0.5 ± 0.1*	1.08 ± 0.1	1.4 ± 0.3	**2.9 ± 0.7**
3KCS4-21	3	1.1 ± 0.1	0.95 ± 0.1	**1.7 ± 0.2**	1.18 ± 0.2	1.14 ± 0.1	1.23 ± 0.2	0.81 ± 0.1	0.9 ± 0.1	2.9 ± 0.4*

^a ^one representative clone for each gene is shown. Three independent biological replicates were performed. Value **± **s.e. indicates expression ratio of Treatment/Control after normalization **± **standard error of the mean (n = 3). Genes that differ significantly (student t test p-value less than 0.05) in their expression between the treatment and control are marked with an asterisk (*). Bold represents expression ratios (≥1.5 or ≤0.6). Underlined values show the opposite regulation on different fluences. The functional categories are 1: protein metabolism/binding, 2: defence, 3: metabolism, 4: Unclassified. Continuous 1: 0.45 μmol m^-2 ^s^-1^; Continuous 2: 0.1 μmol m^-2 ^s^-1^; Pulse: 30 μmol m^-2 ^s^-1 ^for 15 sec. The values shown represent fold change (treatment vs. control).

## Discussion

In this study, we constructed a Ra-specific subtraction library and further selected 33 genes based on the expression analysis to analyse their functional correlation with the early blue-light response. The existence of highly specific genes and low percentages of housekeeping genes indicate that the subtraction was effectively performed for the Ra^+^-Ra^- ^library. Since SSH is expected to normalise the DNA population, the less prevalent genes and highly redundant genes contained in the Ra^+^-Ra^- ^library indicate that the normalisation was successful. The abundance of functionally annotated genes and, in particular, the high proportion of genes related to signalling suggests that Ra activates distinct genetic networks. This high subtractive efficiency allowed us to monitor the expression of many genes in different biochemical or physiological pathways in which Ra is implicated.

### Does raphanusanin modulate cellular defence?

The molecular evidence reported herein indicates that raphanusanin (Ra) is involved in several metabolic processes in which many defence-associated genes are up-regulated and very few genes are down-regulated (Table 3 and Figure 3). Accordingly, questions arise with regard to why Ra activates many defence-associated genes and how this is associated with cellular defence. Ra induced the regulation of leucine-rich repeat (LRR) proteins, LRR transmembrane proteins, the receptor-like kinase (ERECTA), serine-threonine kinases, and glycoproteins (Table 2). Several studies have emphasised the conserved functions of LRR domains, LRR transmembrane proteins [47], membrane-spanning glycoproteins such as hydroxyl proline-rich glycoproteins [48], RLKs such as ERECTA [13,49], and cytoplasmic serine-threonine kinases, such as Ca^+ ^inducers. CBL-interacting protein kinase (CIPK1) [16] acted as a resistant (R) protein in the defence system. Van der Biezen and Jones hypothesised that R proteins may function as regulatory adaptors in plant apoptosomes that are activated by pathogen-derived avirulence (Avr) signals [50].

Moreover, Ra-induced genes (Table 2), such as the antioxidant genes NTRA, CAT, and peroxidase, the channel activators CAMTA3, KT, POT, and H+ ATPase, the lignin-associated genes DRP and peroxidase (overlapping functions as antioxidants), the membrane-associated G-protein Rab, and CPOX are involved in the signal transduction network of the HR (hypersensitive response)-related programmed cell death (PCD) in plants [47]. The earliest cellular response upon R activation includes a rapid burst of reactive oxygen species (ROSs), leading to a dramatic increase in oxidation reactions, increased transmembrane ion flux, especially of Ca^+^, K^+ ^and H^+^, the cross-linking of phenolic moieties with cell-wall components and the reinforcement of the plant cell wall (callose and lignins), the transient activation of protein kinases (wound-induced and salicylic acid (SA)-induced kinases), the production of defensins and phytoalexins, the synthesis of resistance (R) proteins, and transcriptional reprogramming [51].

Many reports have highlighted the involvement of ion channels, especially that of Ca^+^, on the plasma membrane during early defence signalling in *Arabidopsis*, tomato, tobacco, and parsley [52-54]. CAMTA 3 mutants attenuated the propagation of a virulent strain of the bacterial pathogen *Pseudomonas syringae *and the fungal pathogen *Botrytis cinerea *during *Arabidopsi*s development [37]. The rapid and sustained increase in Ca^+ ^is necessary for the oxidative burst and hypersensitive cell death facilitated by the plant disease resistance gene, RPM1, in *Arabidopsis *[55]. Subsequently, the production of ROSs leads to cell-wall fortification, the induction of defence gene expression, and PCD [55,56]. The NADPH thioredoxin (NTR) is involved in the signalling associated with apoptosis and hypersensitivity to stress in *Arabidopsis *[57,58]. The loss of function in the *Arabidopsis *double mutant *ntra ntrb *plant results in hypersensitivity to buthionine sulfoximine (BSO), a specific inhibitor of glutathione biosynthesis, demonstrating the involvement of ntr genes in the glutathione pathway [59]. Moreover, DRP has been suggested to play a role in defence responses and in promoting lignin deposition in juglone-stressed soybeans [15]. Peroxidase is associated with the catalytic reactions of H_2_O_2_, and many studies demonstrate the peroxidase-reduced cell-wall extensibility and elevated lignin content in stressed plants [60-65]. Furthermore, *Arabidopsis *lesion initiation 2 (LIN2) encodes CPOX, and the *lin2 *mutant develops lesions on its leaves and siliques in a developmentally regulated and light-dependent manner [27].

The abundance of genes involved in protein metabolism is remarkable, especially those genes comprising the ubiquitin-proteosome system, HSP90, PMF1, CSN3, UBQ3, CUL1, KEG (a Novel RING E3), and C_3_HC_4_-type Ring Finger. The ubiquitin-proteosome pathway is likely to be an important modulator of the R-gene-triggered resistance [47]. One study showed that one regulator of R-genes, SGT1 (suppressor of G2 allele of SKP1), is essential for the function of the Skp1-cullin-F-box protein (SCF) E3 ubiquitin ligase complex that targets proteins for degradation by the 26S proteosome. RAR1 (required for Mla-dependent resistance1), SGT1, and HSP90 are thought to form a complex that mediates the folding of R proteins into functional complexes [66]. In addition, the COP9 signalosome, a multiprotein complex involved in the recognition of correct substrates for protein degradation, is required for the resistance to tobacco mosaic virus mediated by the tobacco TIR-NB-LRR N protein [67]. Moreover, RING-finger E3 ligases in *Arabidopsis *are involved in the RPM1- and RPS2-mediated elicitation of HR [68]. The knock-down of RING1 transcripts with an artificial microRNA (amiR-R1^159^) leads to hyposensitivity to the pathogenic toxin fumonisin B1 (FB1), whereas over-expression of RING1 confers hypersensitivity [69]. Furthermore, *Arabidopsis *CUL1 (a subunit of E3 ligase) was assembled into the SCF complex containing COI1, an F-box protein required for the response to jasmonates (JA), which regulate plant fertility and defence responses [70], and KEG, an E3 ligase that acts as a negative regulator of abscisic acid (ABA) signalling in *Arabidopsi*s. The *KEG *mutant undergoes growth arrest immediately after germination, suggesting an increase in ABA signalling that regulates the plant survival in unfavourable conditions [30].

Moreover, Ra positively regulated MBP1 (myrosin-binding protein or jasmonate-inducible protein), 1-aminocyclopropane-1-carboxylic acid oxidase (ACCO) (ethylene-forming enzyme), and CAMTA3 (also called ethylene-forming calmodulin-binding protein 1), also correlating with R-gene functions in the hormonal control of PCD activation [47]. The hormone ethylene (ET) is involved in stimulating developmental and inducible forms of PCD during aerenchyma formation [71]. ET positively contributes to HR cell death and lesion size in TMV-infected tobacco leaves, as well as in tomato plants challenged with an avirulent *Xanthomonas *strain [72,73]. Moreover, infection with TMV activates the SIPK cascade (a tobacco mitogen-activated protein kinase MAPK) and induces ethylene biosynthesis. It also induces ACC oxidase [38] because pest-induced wounding increased the ACC oxidase protein in the conifer stem, whereas the methyl jasmonate (MJ) treatment produced a higher and more rapid ACC oxidase response, indicating the coordinated action of ET and JA in defence [12]. Furthermore, JA has been shown to promote cell death events induced by singlet oxygen in the protoplasts of the conditional *flu *mutant in *Arabidopsis *[74] and to affect hairpin-induced hypersensitive cell death in tobacco suspension-cultured BY-2 cells [75]. Genes encoding myrosinase-binding proteins (MBPs) were shown to be H_2_O_2_-responsive [76], and H_2_O_2 _induces PCD in *Arabidopsis *and other species [77-80].

Ra has also been shown to induce the regulation of the phosphatidic acid precursors, phospholipase C (PLC) and phospholipase D (PLD), both of which modulate the PCD as lipid-based signals. For example, the expression of the phosphoinositide-specific phospholipase C gene, OsPI-PLC1, was activated in rice cells after benzothiadiazole (BTH) treatment and in BTH-treated cells after Xanthomonas oryza pv. oryza (Xoo) infection, resulting in the production of an oxidative burst and hypersensitive cell death [17]. The coactivation of PLC, PLD, and ET induces PCD in tomato, and inhibitors of the PLC and PLD signalling pathway intermediates greatly reduce the chemical-induced cell death of suspension-cultured tomato (*Lycopersicon esculentum *Mill.) cells (line MsK8). Ethylene, while not inducing cell death when applied alone, stimulates chemical-induced cell death, indicating that the activation of the PLC, PLD, and ET signalling pathways is required for cell death [18].

The primary role of Ra in hypocotyl growth inhibition, as demonstrated by Moehninsi et al. [3], is likely due to cell-wall strengthening via the induction of lignin to protect cells from environmental stimuli. Alternatively, the interactions between defence and growth suppression could also be a main reason for induction of genes involved in both defence and growth suppression signalling. In support of this notion, one study shows that the indirect activation of the MAPK cascade in H_2_O_2_-treated *Arabidopsis *protoplasts induced the expression of genes involved in defence against oxidative stress and suppressed those associated with growth [81]. In our Ra^+^-Ra^- ^library, four genes thought to be implicated in the functional roles of growth inhibition, DRP, peroxidase, rapid alkalinisation factor 23 (RALF23), and CSN3, were up-regulated in response to Ra. RALF23 is associated with defence via its activation of MAPKs and the induction of medium alkalinisation, leading to growth arrest [19,82]. Moreover, a rapid transient up-regulation of CSN3 is observed in response to various growth inhibition stimuli [3]. In addition, the up-regulation of cellular biogenesis genes such as actin, profilin, cellulose synthase 5-transferase, kinesin, and α-tubulins may also be involved in elaborate cell-wall thickening.

### Raphanusanin-induced gene networks associated with early blue-light (BL) signalling

An attempt to identify the functional correlations between Ra and light regulation led us to monitor the expression of many genes in response to different fluences of early BL. We found that the selected 33 genes were commonly regulated under BL in an intensity or duration-dependent manner (Figure 4 and Table 4). In addition, most gene expression patterns largely overlapped between the BL and exogenous Ra treatments. The above results contribute to an emerging body of evidence indicating that Ra may be functionally correlated with early BL, and thereby affects the regulation of genes involved in establishing plant acclimation to light.

Early blue light effects include the hyperpolarisation of the cell membrane, increased input resistance, and induction of carotenoid biosynthesis, all of which can be observed within the first 30 min after the exposure to light. In addition, light-induced mRNA synthesis of some fast light-regulated genes can be detected within five min after a light pulse [83-89]. Our data are in agreement with the above findings that many genes are induced within the first 30 min, including membrane transporters and many defence-associated and chloroplast-associated genes. The overlapping expression of many genes, especially those with conserved functions involved in early light responses between BL and Ra, indicates that BL and Ra may use a similar signalling mechanism. Many studies show that a rapid phot1-dependent Ca^2+ ^concentration increase occurs in response to blue-light exposure [90-93], thereby triggering the inhibition of hypocotyl growth [94,95]. In addition, the proton extrusion of the phot1-mediated stomatal opening is mediated by an H^+^-ATPase or proton pump, which is triggered by phosphorylation [96-98]. Furthermore, Pedmale and Liscum demonstrated the links between *phot*1, NPH3, and E3 based-ubiquitin-dependent proteosomal degradation in light-mediated processes [99]. The transporters (especially POT, and H^+^-ATPase), membrane bound kinases, E3 ligases, and genes that trigger Ca^2+ ^influx, such as 1, 4, 5-IP3-catalyzing enzyme PLC, CAMTA3, and CIPKI, are differentially regulated in response to both BL and Ra. In addition, under low-BL fluence rates, phot1 is the principle photoreceptor regulating both growth inhibition and phototropism [100-102]. With regard to our experimental system, the ability of unilateral irradiation with low light (LL) fluences of BL to inhibit growth is expected to be under PHOTs control. Taken together, the differential expression of genes induced by BL in this context is likely to be triggered by PHOTs, and the unique signalling cascades triggered by PHOTs may reflect crosstalk between the light and defence systems. This notion is supported by a report showing that the FMN moiety of PHOT1 confers redox sensitivity that leads to its activation [103,104]. Many previous studies demonstrated BL-induced gene expression profile changes using experimental conditions that include high light (HL) and long time-exposure [105-107]. Our data showing gene expression profiles within the first 30 min under LL intensities are informative with regard to the signalling pathways responsive to variable fluence rates and early light responses while plants are acclimating to sudden environmental changes. This information may be useful for identifying the photosensory signalling networks that interact with cellular defence.

### Raphanusanin may mediate the links between the light signalling and defence responses

Because Ra induces the up-regulation of many defence-related genes, the obvious question is how light affects the Ra-induced signalling cascades involved in the induction of transcription factors and their target genes. Earlier studies have pointed out that light not only modulates the defence responses via its influence on biochemistry and plant development, but is also essential for the development of resistance [108]. For examples, light is necessary for development of the resistance responses to *Pseudomonas solanacearum *in tobacco (*Nicotiana tubacum*) [109], *Xanthomonas oryzae *in rice (*Oryza sativa*) [110], and *Pseudomonas syringae *and *Peronospora parasitica *in Arabidopsis [111,112]. There are also several examples of plant responses to isolated pathogenic elicitors that are light-dependent. Leaf necrosis on tomato in response to an avirulence elicitor from *Cladosporium fulvum *is substantially reduced in the dark [113], cell death induced by the fungal toxins AAL from *Alternaria alternata *[114] requires light, and one of the pathogenic toxins (FB1) capable of inducing cell death in Arabidopsis protoplasts is light-dependent and requires phytohormones, as well as the SA, JA, and ET-mediated signalling pathways [115]. The importance of the cellular energy status and redox balance (H_2_O_2_/ROS) produced in chloroplasts and plant stress responses in the regulation of PCD is supported by the finding that various forms of cell death triggered by pathogens, or spontaneously in lesion-mimic mutants, are light-dependent [88,110,116-119]. Additionally, at least some of the pathways involved in the biosynthesis of the major-related hormones, JA, SA, and ABA, are located in the chloroplast, thus revealing a role for photosynthesis in both abiotic and biotic stress responses [87,108]. Considering that, many Ra-induced genes involved in the predicted PCD pathways may simply reflect the light-dependent manner of PCD, and the roles of Ra in cellular defence may be dependent upon light.

## Conclusions

This is the first report on comprehensive survey of transcriptional regulation in response to the light-induced growth inhibitor, Ra. Although physiological evidence for it roles in the inhibition of hypocotyls growth, microtubule orientation, and inhibition of apical dominance have been demonstrated, the molecular mechanisms underlying regulation by Ra are still limited. Our data offer the comprehensive understanding of transcriptional regulation in etiolated radish seedlings in response to Ra and its functional correlation with BL. We also provide a number of genes that are regulated in response to Ra that could be tested in many functional analyses to increase our understanding of the roles of Ra. Most of the genes important for cellular defence are highly regulated by both Ra and BL. These results suggest the link between Ra and cellular defence and light signalling. Further research on the biological relevance of the effects of Ra on plant-light-microbe interactions and the analysis of null mutants in specific pathways should provide new insights into the role of Ra in cellular defence.

## Methods

### Plant material and plant growth

Sakurajima radish (*Raphanus sativus *var. *hortensis *f. *gigantissimus *Makino) seeds were germinated in vermiculite moistened with water in large trays (37 × 60 × 14 cm) in absolute darkness at 25°C. About 3 d later, uniform seedlings were transplanted to small trays (8.5 × 17.7 × 3.5 cm) containing moist vermiculite under extremely dim green 'safelight' (<0.01 μmol m^-2 ^s^-1^) and incubated in the dark at 25°C for 1 d.

### Light treatments and unilateral application of raphanusanin

Light treatments were performed using an LED array (NSPB 520S; NICHIA for blue light (BL). Etiolated seedlings (with a hypocotyl length of about 4 cm) were unilaterally illuminated with BL (LED array; m_ix_: 470 nm; half band width: 20 nm) for 30 min at 25°C. The incident energy was 0.45 μmol m^-2 ^s^-1^, 0.1 μmol m^-2 ^s^-1^, or a pulse (30 μmol m^-2 ^s^-1 ^for 10 sec) at plant level. Fluence rates were assessed with a LI-COR LI-189 photometer. Raphanusanin was isolated from fresh radish roots based on the procedures described by Kosemura et al., 1997. An estimated endogenous amount of 50 ng of Ra was mixed with 0.5 mg of lanolin and unilaterally applied to the hypocotyls in a lengthwise manner from 0 to 2 cm below the hook of uniform four-day old etiolated seedlings. Control seedlings were also treated with 0.5 mg lanolin. Treated seedlings were incubated in absolute darkness at 25°C. All manipulations were performed under safelight (>0.01 μmol m^-2 ^s^-1^).

### Sample collection

Etiolated four-day-old seedlings were unilaterally illuminated with BL (0.45 μmol m^-2 ^s^-1^, 0.1 μmol m^-2 ^s^-1 ^or pulse 30 μmol m^-2 ^s^-1 ^for 10 sec) or treated with a unilateral application of raphanusanin (50 ng) using the same procedure mentioned above. The seedlings were harvested and immediately submerged in liquid nitrogen with minimal exposure to 'safelight' after the indicated time periods. Treated samples were immediately replaced in the dark box for 5, 15, or 30 min until harvested. Ten replicates (both control and treated samples) were harvested directly into liquid nitrogen after the following treatments.

### Subtraction library construction

Suppression subtractive hybridization (SSH) was performed using a PCR-select cDNA subtraction kit (Clontech Laboratories, U.S.A.) according to the manufacturer's instructions (see detailed in additional file 1 and additional file 6).

### Determination of nucleotide sequences and sequence annotation

Nucleotide sequences were determined with a DNA autosequencer (ABI 310 Applied Biosystems, USA) using Big-Dye terminators. All sequencing reactions involved either the standard M13 forward or reverse primers, and thus both the 5' and 3' sequences of each cDNA were obtained. The sequence text files were edited to remove vector sequence and ambiguous bases. Two reads from both ends of a clone were merged using the Codon Code Aligner based on pairwise alignments. The resulting sequences were then assembled by the Phrap program (Codon Code Aligner Sequence Assembler v3.0.1). The annotation is based on the best BLASTX match of the corresponding radish sequences against NCBI non-redundant protein sequences (nr) (expect value < 0.01) or TAIR Arabidopsis protein database. Physiological and biochemical classification of the clones were clustered according to GO annotations http://www.ebi.ac.uk/GOA/.

### Selection of radish sequences and PCR primer design

To identify the true reference gene for evaluating the gene expression level of raphanusanin-induced clones, seven housekeeping genes commonly used as controls for plant gene expression studies, elongation factor 1-α (ef1α), translation initiation factor (eIf2), 18s rRNA, actin, tubulin, ubiquitin and ribosomal protein (L4), were selected. Radish nucleotide sequences for tubulin, eIf2, ubiquitin, and ribosomal protein (L4) were obtained from the sequences of radish-subtracted library clones. The sequences of 18s rRNA and actin were obtained from radish ESTs deposited in the Gene bank database. The only ef1α sequence found was from *Arabidopsis thaliana*, and the conserved region of this gene was selected for primer design. Seven primer pairs were designed based on these sequences for reference gene analysis using Primer3 software (see additional file 7). Fifty primer pairs for expression analysis of Ra-induced clones were designed based on the sequences of raphanusanin-induced ESTs using Primer3 software (see additional file 8). BLASTX searches were performed against the sequence databases to confirm the gene specificity of the primer sequences.

### Quantitative real-Time RT-PCR

Total cellular RNA was extracted using a plant RNeasy Mini kit (QIAGEN, Germany) according to the manufacturer's instructions, followed by removal of contaminating genomic DNA with an RNase-Free DNase Set (QIAGEN, Germany). The first strand of cDNA was then synthesized using ThermoScript RNase H^- ^RT (Invitrogen, USA) with an Oligo (dT)_12-18 _primer (Invitrogen, USA). Three independent preparations of mRNA for each biological replicate were pooled to eliminate the inconsistent variation with sampling. Quantitative real-time PCR (qRT-PCR) was performed with a Thermal Cycler Dice™ Real Time System (TAKARA BIO INC., Japan) using SYBR^® ^Premix Ex Taq™ II (perfect real time) (TAKARA BIO INC., Japan). Reactions were performed in a total volume of 20 ul containing 1×SYBR Premix Ex Taq II, 25 ng of cDNA, 200 nM of each specific sense and antisense primer, except for 18s rRNA primers, for which 50 nM of each was used. The two-step amplification program was: 95°C for 10 sec, 45 cycles of 95°C for 5 s, followed by 61°C for 45 s. Each sample had 2 replicates and non-template control to ensure reproducibility of the results. The real-time PCR efficiency was determined for each gene and each treatment via standard curve analysis. For this, each cDNA sample was pooled and then used as the PCR template (range of 50, 25, 12.5, 7.5, and 3.75 ng). All PCR reactions displayed efficiencies between 88% and 105%. The specificity of the amplification was verified by both dissociation curve analysis and by visualization via gel electrophoresis. The most stable gene, eIf2 or ef1α, resulting from the control gene analysis was used as a reference gene. Relative expression levels were calculated using the comparative C_T _method. For each gene, expression values were normalized to the control samples (time zero), which were set to equal 1. Each value represents the average of three experimental replications. Within a single experiment, aliquots of the same cDNA synthesis reaction were used for real-time PCR amplification of each of the seven genes and all gene primers and cDNA combinations were amplified in duplicate in a single PCR run.

### RT-PCR detection

The preparation of total RNA and first strand cDNA synthesis were performed as above. The PCR amplification was carried out in a 20 μl reaction volume containing 100 ng of cDNA as template, 1 × PCR buffer, 20 μM dNTPs, 2.5 mM MgCl_2_^+^, 0.4 μM primers, and 0.5 U of HS (Hot Start) Taq polymerase (Takara, Japan). The number of cycles used for the PCR reaction was adjusted for each gene to obtain barely visible bands by agarose gel electrophoresis. The PCR conditions were as follows: 95°C for 3 min, followed by the indicated number of cycles at 94°C for 30 s, 61°C for 30 s, and 72°C for 45 s. A final extension was carried out at 72°C for 5 min. A 15 μl aliquot of each PCR product was electrophoresed in a 3.5% w/v agarose gel. The primer pairs used for each gene were the same as those used for qRT-PCR.

### Statistical analyses

Results (C_T values) _from the Thermal Cycler Dice™ Real Time System were analyzed in Microsoft Excel. The levels present in different samples were calculated by F statistics [F = between tissue sample mean square/error mean square]. Other statistics defined in Table 2 were calculated using the method of Brunner et al., 2003 [120].

## Authors' contributions

MHS designed experiments, acquisition of data, compiled results and wrote the manuscript. KM carried out the organization of the data and manuscript editing. HN provided assistance in some experiments. KY, KH and HS directed the project. All authors read and approved the final manuscript.

## Supplementary Material

Additional file 1 Figure S1: Determination of subtraction efficiency. Click here for file

Additional file 2 Table S1: Physiological characterization of gene clusters Click here for file

Additional file 3 **Table S2: Biochemical characterization of gene clusters**. Click here for file

Additional file 4 Figure S2: RNA transcription levels of housekeeping genes tested in the raphanusanin-treated sample, presented as the C_T _mean value at different time points. Click here for file

Additional file 5 Table S3: Summary of statistics pertaining to the stability of gene expression. Click here for file

Additional file 6 **Materials S1**. Construction of subtraction library Click here for file

Additional file 7 Table S4: Primer sequences of seven housekeeping genes, the amplification length and the melting temperature of the amplified product. Click here for file

Additional file 8 Table S5: Primer sequences of fifty ESTs, the amplification length and the melting temperature of the amplified product. Click here for file
