# Supplementary material for: Comparative transcriptional profiling-based identification of raphanusanin-inducible genes
Source: BMC Plant Biol. 2010 Jun 16;10:111. doi: 10.1186/1471-2229-10-111 (PMC3095276; doi:10.1186/1471-2229-10-111)
Supplement: Additional file 1 — Figure S1: Determination of subtraction efficiency. [file 1471-2229-10-111-S1.DOC]

**Additional file 1**

**Figure S1**

Figure S1: Determination of subtraction efficiency. (A) Digested and Undigested double stranded cDNA products. Undigested double stranded cDNA products (lane 1) and *Rsa*I digested double stranded cDNA products(lane 2). 100 bp marker (lane M). **(**B) Detection of adaptor ligation efficiency. Tester1-1 was amplified with 18S rRNA 3’, PCR primer1 (lane 1) and tester1-1 with 18S rRNA3’ and 5’ primers (lane 2). Tester1-2 was amplified with 18Ss rRNA 3’ primer, PCR primer1 (lane 3) and tester1-2 with 18S rRNA 3’ and 5’ primers (lane 4). 100bp size marker (lane M). (C) Analyses of PCR products. Secondary PCR products of subtracted samples (lane 1) and unsubtracted samples (lane 2). ɸX174 DNA/*Hae* III digest size markers (lane M). (D) Identification of subtraction efficiency by PCR. 18S rRNA PCR products of subtracted samples at 18, 23, 28, and 33 cycles (lanes 1, 2, 3, 4) respectively and 18S rRNA PCR products of unsubstracted samples respectively at 18, 23, 28, 33 cycles (Lanes 5, 6, 7 and 8). 100 bp size marker (lane M)
