# Supplementary material for: Comparative transcriptional profiling-based identification of raphanusanin-inducible genes
Source: BMC Plant Biol. 2010 Jun 16;10:111. doi: 10.1186/1471-2229-10-111 (PMC3095276; doi:10.1186/1471-2229-10-111)
Supplement: Additional file 2 — Table S1: Physiological characterization of gene clusters [file 1471-2229-10-111-S2.DOC]

**Additional file 2**

**Table S1**

Table S1: Physiological characterization of gene clusters.

| **Clone ID** | **Accession No.** | **Locus** | **Size (bp)** | **E-value** | **Description and putative function closest to database match** |
| --- | --- | --- | --- | --- | --- |
|  |  |  |  |  | **Defense related** |
| Rs-Ra002 | AB447895 | AT5G16590 | 177 | 7.00E-17 | [ref|NP_197162.1|](http://www.ncbi.nlm.nih.gov/entrez/query.fcgi?cmd=Retrieve&db=Protein&list_uids=15237379&dopt=GenPept&RID=SJ83M47N012&log$=protalign&blast_rank=1) LRR1; ATP binding / kinase/ protein serine/threonine kinase [Arabidopsis thaliana] |
| Rs-Ra003 | AB447896 | [AT1G56145](http://www.ncbi.nlm.nih.gov/sites/entrez?db=gene&cmd=search&term=842067&RID=4YYBENM3012&log$=geneexplicitprot&blast_rank=3) | 293 | 9.00E-44 | [ref|NP_564710.1|](http://www.ncbi.nlm.nih.gov/entrez/query.fcgi?cmd=Retrieve&db=Protein&list_uids=18405703&dopt=GenPept&RID=SJ8JY63N016&log$=protalign&blast_rank=3) leucine-rich repeat family protein [Arabidopsis thaliana] |
| Rs-Ra006 | AB447899 | AT2G30360 | 549 | 4.00E-30 | [ref|NP_180595.1|](http://www.ncbi.nlm.nih.gov/entrez/query.fcgi?cmd=Retrieve&db=Protein&list_uids=15227739&dopt=GenPept&RID=SJ9KGYR9014&log$=protalign&blast_rank=1)SNF 1-related protein kinase (CIKP1) [Arabidopsis thaliana] |
| Rs-Ra007 | AB447900 | [AT2G26330](http://www.ncbi.nlm.nih.gov/entrez/query.fcgi?db=gene&cmd=Retrieve&dopt=full_report&list_uids=817173) | 309 | 1.00E-30 | [ref|NP_180201.1|](http://www.ncbi.nlm.nih.gov/entrez/query.fcgi?cmd=Retrieve&db=Protein&list_uids=15225286&dopt=GenPept&RID=SJ9WSD9K012&log$=protalign&blast_rank=2)receptor protein kinase (ERECTA) [Arabidopsis thaliana] |
| Rs-Ra005 | AB447898 | AT4G03260 | 472 | 3.00E-43 | [ref|NP_192235.3|](http://www.ncbi.nlm.nih.gov/entrez/query.fcgi?cmd=Retrieve&db=Protein&list_uids=30679417&dopt=GenPept&RID=SJBESYUH016&log$=protalign&blast_rank=2)Leucine-rich repeat family protein[Arabidopsis thaliana] |
| Rs-Ra014 | AB447907 | AT3G16785 | 477 | 1.00E-55 | [ref|NP_188302.2|](http://www.ncbi.nlm.nih.gov/entrez/query.fcgi?cmd=Retrieve&db=Protein&list_uids=22331122&dopt=GenPept&RID=SJD55THA01N&log$=protalign&blast_rank=1)PLDP1 (phospholipase D Zeta 1) [Arabidopsis thaliana] |
| Rs-Ra001 | AB447894 | [AT5G53570](http://www.ncbi.nlm.nih.gov/sites/entrez?db=gene&cmd=search&term=835439&RID=73748WUH012&log$=geneexplicitprot&blast_rank=2) | 352 | 3.00E-14 | [ref|NP_001154777.1|](http://www.ncbi.nlm.nih.gov/entrez/query.fcgi?cmd=Retrieve&db=Protein&list_uids=238481558&dopt=GenPept&RID=SJ7JG4WX012&log$=protalign&blast_rank=1) Rab-like small GTPases-like protein [Arabidopsis thaliana] |
| Rs-Ra038 | AB447931 | [AT5G13530](http://www.arabidopsis.org/servlets/TairObject?type=locus&name=AT5G13530) | 389 | 2.00E-12 | [ref|NP_196857.2|](http://www.ncbi.nlm.nih.gov/entrez/query.fcgi?cmd=Retrieve&db=Protein&list_uids=186522588&dopt=GenPept&RID=SK17UM2V01S&log$=protalign&blast_rank=2)RING E3 ligase protein (KEG) [Arabidopsis thaliana] |
| Rs-Ra017 | AB447910 | AT4G24190 | 396 | 6.00E-27 | [gb|AAB63606.1|](http://www.ncbi.nlm.nih.gov/entrez/query.fcgi?cmd=Retrieve&db=Protein&list_uids=2262098&dopt=GenPept&RID=SJDNDVD101S&log$=protalign&blast_rank=1) SHD (SHEPHERD) ATP binding/HSP90[Arabidopsis thaliana] |
| Rs-Ra032 | AB447925 | Y11482 | 339 | 4.00E-38 | [gb|AAC08049.1|](http://www.ncbi.nlm.nih.gov/entrez/query.fcgi?cmd=Retrieve&db=Protein&list_uids=1655826&dopt=GenPept&RID=SK06MHXR01S&log$=protalign&blast_rank=1)Myrosinase binding protein (MBP1)/ (Jacalin-like lectin domain) [Brassica napus] |
| Rs-Ra039 | AB447932 | AT4G02570 | 295 | 1.0E-25 | [ref|NP_567243.1|](http://www.ncbi.nlm.nih.gov/entrez/query.fcgi?cmd=Retrieve&db=Protein&list_uids=18411983&dopt=GenPept&RID=SK1C6TZJ012&log$=protalign&blast_rank=2)CULLIN 1, a subunit of E3 ubiquitin ligase (CUL1) [Arabidopsis thaliana] |
| Rs-Ra041 | AB447934 | AT5G03240 | 320 | 2.00E-23 | [ref|NP_851029.1|](http://www.ncbi.nlm.nih.gov/entrez/query.fcgi?cmd=Retrieve&db=Protein&list_uids=30679945&dopt=GenPept&RID=SK1N96SP012&log$=protalign&blast_rank=10)Ubiquitin (gene=UBQ3) [Arabidopsis thaliana] |
| Rs-Ra024 | AB447917 | [AT3G16570](http://www.ncbi.nlm.nih.gov/sites/entrez?db=gene&cmd=search&term=820907&RID=6KFM6D6R016&log$=geneexplicitprot&blast_rank=1) | 130 | 3.00E-10 | RALF 23 (LILE 23) [Arabidopsis thaliana] |
| Rs-Ra026 | AB447919 | [AT3G53990](http://www.ncbi.nlm.nih.gov/entrez/query.fcgi?db=gene&cmd=Retrieve&dopt=full_report&list_uids=824566) | 600 | 4.00E-75 | [ref|NP_566991.2|](http://www.ncbi.nlm.nih.gov/entrez/query.fcgi?cmd=Retrieve&db=Protein&list_uids=30693971&dopt=GenPept&RID=SJFD942201S&log$=protalign&blast_rank=2) Universal stress protein (USP) family protein [Arabidopsis thaliana] |
| Rs-Ra034 | AB447927 | [AT4G23690](http://www.ncbi.nlm.nih.gov/sites/entrez?db=gene&cmd=search&term=828469&RID=61UKBTTP01R&log$=geneexplicitprot&blast_rank=4) | 385 | 1.00E-15 | [ref|NP_194100.1|](http://www.ncbi.nlm.nih.gov/entrez/query.fcgi?cmd=Retrieve&db=Protein&list_uids=15236570&dopt=GenPept&RID=SK0GASJU01N&log$=protalign&blast_rank=3) Disease resistence response protein (DRP) [Arabidopsis thaliana] |
| Rs-Ra022 | AB447915 | AB042187 | 562 | 1.00E-86 | [dbj|BAB17227.1|](http://www.ncbi.nlm.nih.gov/entrez/query.fcgi?cmd=Retrieve&db=Protein&list_uids=11034736&dopt=GenPept&RID=SJEF7J5F01N&log$=protalign&blast_rank=1) Myrosinase (RMB2) [Raphanus sativus] |
| Rs-Ra022 | AB447915 | AB04218 | 199 | 7.00E-10 | [dbj|BAB17226.1|](http://www.ncbi.nlm.nih.gov/entrez/query.fcgi?cmd=Retrieve&db=Protein&list_uids=11034734&dopt=GenPept&RID=SJEG8T2S014&log$=protalign&blast_rank=1) Myrosinase (RMB1) [Raphanus sativus] |
| Rs-Ra020 | AB447913 | AF139538 | 630 | 2.00E-90 | [AAD30291.2](http://www.ncbi.nlm.nih.gov/entrez/query.fcgi?cmd=Retrieve&db=Protein&list_uids=6563394&dopt=GenPept&RID=SJE3AFUV01S&log$=prottop&blast_rank=1) Catalase2 (CAT) [Raphanus sativus] |
| Rs-Ra028 | AB447921 | X81628.1 | 353 | 4.00E-50 | [emb|CAA57284.1|](http://www.ncbi.nlm.nih.gov/entrez/query.fcgi?cmd=Retrieve&db=Protein&list_uids=587086&dopt=GenPept&RID=SJZDTP0E01S&log$=protalign&blast_rank=1) ACC oxidase (ACCO) [Brassica oleracea] |
| Rs-Ra036 | AB447929 | AT3G32980 | 413 | 2.00E-45 | [ref|NP_850652.1|](http://www.ncbi.nlm.nih.gov/entrez/query.fcgi?cmd=Retrieve&db=Protein&list_uids=30690396&dopt=GenPept&RID=SK0UH49Z01S&log$=protalign&blast_rank=5)Peroxidase[Arabidopsis thaliana] |
| Rs-Ra033 | AB447926 | AT2G17420 | 334 | 3.00E-39 | [ref|NP_179334.4|](http://www.ncbi.nlm.nih.gov/entrez/query.fcgi?cmd=Retrieve&db=Protein&list_uids=79557518&dopt=GenPept&RID=SK0AEMX101S&log$=protalign&blast_rank=4)NTRA (NADPH-dependent thioredoxin reductase 2) [Arabidopsis thaliana] |
| Rs-Ra018 | AB447911 | AT5G14520 | 547 | 2.00E-80 | [ref|NP_196956.1|](http://www.ncbi.nlm.nih.gov/entrez/query.fcgi?cmd=Retrieve&db=Protein&list_uids=15241421&dopt=GenPept&RID=SJDV5X1K01S&log$=protalign&blast_rank=1)Pescadillo-related protein (Pescadillo) [Arabidopsis thaliana] |
| Rs-Ra025 | AB447918 | AT2G22300 | 502 | 1.00E-73 | [dbj|BAE98628.1|](http://www.ncbi.nlm.nih.gov/entrez/query.fcgi?cmd=Retrieve&db=Protein&list_uids=110741068&dopt=GenPept&RID=SJET8RMX012&log$=protalign&blast_rank=1) Calmodulin-binding transcription activator 3 (CAMTA3) [Arabidopsis thaliana] |
| Rs-Ra035 | AB447928 | AT1G20840 | 463 | 3.0E-46 | [ref|NP_173508.1|](http://www.ncbi.nlm.nih.gov/entrez/query.fcgi?cmd=Retrieve&db=Protein&list_uids=15218044&dopt=GenPept&RID=SK0P16AE01N&log$=protalign&blast_rank=3)TMT1(Tonoplast monosaccharides transporter1) [Arabidopsis thaliana] |
| Rs-Ra078 | AB447971 | AT3G18780 | 151 | 3.00E-10 | [ref|NP_175350.1|](http://www.ncbi.nlm.nih.gov/entrez/query.fcgi?cmd=Retrieve&db=Protein&list_uids=15222075&dopt=GenPept&RID=SKEDB0TD01S&log$=protalign&blast_rank=5) Actin8/ light stress-regulated 2[Arabidopsis thaliana] |
| Rs-Ra031 | AB447924 | AT2G39750 | 457 | 2.00E-58 | [ref|NP_030521.1|](http://www.ncbi.nlm.nih.gov/entrez/query.fcgi?cmd=Retrieve&db=Protein&list_uids=18405149&dopt=GenPept&RID=SK023Z4R01N&log$=protalign&blast_rank=1)Dehydration-responsive family protein (Dehydrin) [Arabidopsis thaliana] |
| Rs-Ra075 | AB447968 | [AT5G22580](http://www.ncbi.nlm.nih.gov/sites/entrez?db=gene&cmd=search&term=832321&RID=65ZBMEMU016&log$=geneexplicitprot&blast_rank=1) | 541 | 1.00E-37 | [ref|NP_568422.1|](http://www.ncbi.nlm.nih.gov/entrez/query.fcgi?cmd=Retrieve&db=Protein&list_uids=18420541&dopt=GenPept&RID=SK7JCJRY014&log$=protalign&blast_rank=1) Unknown protein/Stress responsive A/B Barrel Domain[Arabidopsis thaliana] |
| Rs-Ra079 | AB447972 | AT5G09870 | 174 | 2.00E-25 | [ref|NP_196549.1|](http://www.ncbi.nlm.nih.gov/entrez/query.fcgi?cmd=Retrieve&db=Protein&list_uids=15242540&dopt=GenPept&RID=SKA1ENSS01S&log$=protalign&blast_rank=1)Cellulose synthase 5- transferase (CESA5) [Arabidopsis thaliana] |
| Rs-Ra037 | AB447930 | AT5G17920 | 512 | 2.00E-58 | [ref|NP_197294.1|](http://www.ncbi.nlm.nih.gov/entrez/query.fcgi?cmd=Retrieve&db=Protein&list_uids=15238686&dopt=GenPept&RID=SK13430N014&log$=protalign&blast_rank=4) Cobalamin-independent methionine synthase (MetE) [Arabidopsis thaliana] |
| Rs-Ra027 | AB447920 | AT4G09750 | 240 | 2.00E-07 | [ref|NP_192713.1|](http://www.ncbi.nlm.nih.gov/entrez/query.fcgi?cmd=Retrieve&db=Protein&list_uids=15234031&dopt=GenPept&RID=SJFEXEXX014&log$=protalign&blast_rank=2)short-chain dehydrogenase (SDR) [Arabidopsis thaliana] |
| Rs-Ra029 | AB447922 | AF375424 | 444 | 4.00E-48 | [ref|NP_171847.4|](http://www.ncbi.nlm.nih.gov/entrez/query.fcgi?cmd=Retrieve&db=Protein&list_uids=240254000&dopt=GenPept&RID=SJZNTARH01N&log$=protalign&blast_rank=2)Coproporphyrinogen III oxidase (CPOX) [Arabidopsis thaliana] |
| Rs-Ra059 | AB447952 | [AT5G59290](http://www.ncbi.nlm.nih.gov/entrez/query.fcgi?db=gene&cmd=Retrieve&dopt=full_report&list_uids=836047) | 588 | 2.00E-86 | [ref|NP_001078768.1|](http://www.ncbi.nlm.nih.gov/entrez/query.fcgi?cmd=Retrieve&db=Protein&list_uids=145334845&dopt=GenPept&RID=SK4RPCUG016&log$=protalign&blast_rank=1)UDP-D glucuronate carboxy-lyase[Arabidopsis thaliana] |
| RaiA | AB355981 | AF361759 | 340 | 8.00E-49 | [dbj|BAG11654.1|](http://www.ncbi.nlm.nih.gov/entrez/query.fcgi?cmd=Retrieve&db=Protein&list_uids=168983526&dopt=GenPept&RID=SKEY2JT301S&log$=protalign&blast_rank=1)Cop 9 signalosome subunit 3 (CSN3) [Raphanus sativus] |
| Rs-Ra019 | AB447912 | AT1G19710 | 259 | 9.00E-15 | [ref|NP_173401.1|](http://www.ncbi.nlm.nih.gov/entrez/query.fcgi?cmd=Retrieve&db=Protein&list_uids=15223628&dopt=GenPept&RID=SJDWGG0801N&log$=protalign&blast_rank=2)Glycosyl transferase family 1 protein (GTF) [Arabidopsis thaliana] |
| Rs-Ra030 | AB447923 | AT5G35790 | 216 | 7.00E-25 | [dbj|BAD94743.1|](http://www.ncbi.nlm.nih.gov/entrez/query.fcgi?cmd=Retrieve&db=Protein&list_uids=62321397&dopt=GenPept&RID=SJZY9VVY01N&log$=protalign&blast_rank=1) Glucose 6 -phosphate dehydrogenase (G6PD) [Arabidopsis thaliana] |
| Rs-Ra021 | AB447914 | AT1G14710 | 365 | 1.00E-08 | [ref|NP_563957.1|](http://www.ncbi.nlm.nih.gov/entrez/query.fcgi?cmd=Retrieve&db=Protein&list_uids=18394153&dopt=GenPept&RID=SJE4HZH3016&log$=protalign&blast_rank=3)Hydroxy proline rich glycoprotein family (Glycoprotein) [Arabidopsis thaliana] |
| Rs-Ra084 | AB447977 | AT5G15230 | 389 | 9.00E-23 | [ref|NP_001078587.1|](http://www.ncbi.nlm.nih.gov/entrez/query.fcgi?cmd=Retrieve&db=Protein&list_uids=145334444&dopt=GenPept&RID=SKBTXUZZ016&log$=protalign&blast_rank=1) GASA4 (GAST1 protein homolog) [Arabidopsis thaliana] |
| Rs-Ra008 | AB447901 | AAD26119.1 | 636 | 1.00E-100 | [gb|AAD26119.1|AF108123_1](http://www.ncbi.nlm.nih.gov/entrez/query.fcgi?cmd=Retrieve&db=Protein&list_uids=4588474&dopt=GenPept&RID=SJBZC1BV01N&log$=protalign&blast_rank=1)Phosphoinositide- specific phospholipase C (PLC) [Brassica napus] |
| Rs-Ra004 | AB447897 | [AT2G30740](http://www.ncbi.nlm.nih.gov/sites/entrez?db=gene&cmd=search&term=817625&RID=6T075R6N01R&log$=geneexplicitprot&blast_rank=1) | 517 | 1.00E-74 | [emb|CAC34450.1|](http://www.ncbi.nlm.nih.gov/entrez/query.fcgi?cmd=Retrieve&db=Protein&list_uids=13374083&dopt=GenPept&RID=SJ8ZV90T014&log$=protalign&blast_rank=1) PTI1-like protein tyrosine kinase [Arabidopsis thaliana] |
| Rs-Ra040 | AB447933 | [AT1G67250](http://www.ncbi.nlm.nih.gov/sites/entrez?db=gene&cmd=search&term=843045&RID=4YY38ABF012&log$=geneexplicitprot&blast_rank=1) | 559 | 2.00E-39 | [ref|NP_564892.1|](http://www.ncbi.nlm.nih.gov/entrez/query.fcgi?cmd=Retrieve&db=Protein&list_uids=18408726&dopt=GenPept&RID=SK1FCRSA016&log$=protalign&blast_rank=2)Proteasome maturation factor (UMP1) [Arabidopsis thaliana] |
| Rs-Ra011 | AB447904 | AT2G31880 | 606 | 1.00E-64 | [ref|NP_180747.1|](http://www.ncbi.nlm.nih.gov/entrez/query.fcgi?cmd=Retrieve&db=Protein&list_uids=15225153&dopt=GenPept&RID=SJCPB2Y6014&log$=protalign&blast_rank=2) Leucine-rich repeat tranmembrane protein Kinase[Arabidopsis thaliana] |
| Rs-Ra012 | AB447905 | [AT5G45430](http://www.ncbi.nlm.nih.gov/sites/entrez?db=gene&cmd=search&term=834579&RID=7RZ56STW014&log$=geneexplicitprot&blast_rank=2) | 476 | 2.00E-57 | [gb|AAK96701.1|](http://www.ncbi.nlm.nih.gov/entrez/query.fcgi?cmd=Retrieve&db=Protein&list_uids=15450860&dopt=GenPept&RID=SJCWET1K01S&log$=protalign&blast_rank=1) serine/theorine protein kinase (Mak) [Arabidopsis thaliana] |
| Rs-Ra042 | AB447935 | [AT3G09760](http://www.ncbi.nlm.nih.gov/sites/entrez?db=gene&cmd=search&term=820134&RID=5PTYDFRD015&log$=geneexplicitprot&blast_rank=1) | 628 | 2.00E-62 | [ref|NP_566355.1|](http://www.ncbi.nlm.nih.gov/entrez/query.fcgi?cmd=Retrieve&db=Protein&list_uids=18398630&dopt=GenPept&RID=SK1U7S4P01S&log$=protalign&blast_rank=1) C3HC4-type Ring Finger (Zinc Finger) [Arabidopsis thaliana] |
| Rs-Ra043 | AB447936 | AT4G19960 | 104 | 4.00E-10 | [ref|NP_193729.2|](http://www.ncbi.nlm.nih.gov/entrez/query.fcgi?cmd=Retrieve&db=Protein&list_uids=145340436&dopt=GenPept&RID=SK229DXG016&log$=protalign&blast_rank=1)potassium iron transporter (KT) [Arabidopsis thaliana] |
| Rs-Ra044 | AB447937 | AT3G16180 | 581 | 2.00E-84 | [ref|NP_188239.1|](http://www.ncbi.nlm.nih.gov/entrez/query.fcgi?cmd=Retrieve&db=Protein&list_uids=15233307&dopt=GenPept&RID=SK1XEW7P012&log$=protalign&blast_rank=2)Proton-dependent oligopeptide transport family protein (POT) [Arabidopsis thaliana] |
| Rs-Ra061 | AB447954 | AT3G28710 | 216 | 1.00E-07 | [ref|NP_189512.1|](http://www.ncbi.nlm.nih.gov/entrez/query.fcgi?cmd=Retrieve&db=Protein&list_uids=15233060&dopt=GenPept&RID=SK58C2GK012&log$=protalign&blast_rank=2) H+-transport two-sector ATPase[Arabidopsis thaliana] |
| Rs-Ra048 | AB447941 | [AT3G04950](http://www.ncbi.nlm.nih.gov/entrez/query.fcgi?db=gene&cmd=Retrieve&dopt=full_report&list_uids=819655) | 258 | 7.00E-20 | [ref|NP_187146.2|](http://www.ncbi.nlm.nih.gov/entrez/query.fcgi?cmd=Retrieve&db=Protein&list_uids=79393348&dopt=GenPept&RID=SK2M5ACE016&log$=protalign&blast_rank=1) FfG singnal recognition particle GTPase[Arabidopsis thaliana] |
| Rs-Ra057 | AB447950 | AT3G44260 | 162 | 5.00E-15 | [ref|NP_190012.1|](http://www.ncbi.nlm.nih.gov/entrez/query.fcgi?cmd=Retrieve&db=Protein&list_uids=15229916&dopt=GenPept&RID=SK4ACTD7014&log$=protalign&blast_rank=1) CCR4-NOT transcription complex protein (CCR4-NOT) [Arabidopsis thaliana] |
|  |  |  |  |  | **Metabolism** |
| Rs-Ra008 | AB447953 | [AT1G17745](http://www.ncbi.nlm.nih.gov/entrez/query.fcgi?db=gene&cmd=Retrieve&dopt=full_report&list_uids=838352) | 496 | 1.00E-60 | [ref|NP_564034.1|](http://www.ncbi.nlm.nih.gov/entrez/query.fcgi?cmd=Retrieve&db=Protein&list_uids=18394525&dopt=GenPept&RID=SK4XU8X6012&log$=protalign&blast_rank=1) 3-Phosphoglycerate dehydrogenase (PGDH) [Arabidopsis thaliana] |
| Rs-Ra064 | AB447957 | [AT1G60810](http://www.ncbi.nlm.nih.gov/entrez/query.fcgi?db=gene&cmd=Retrieve&dopt=full_report&list_uids=842375) | 518 | 3.00E-92 | [ref|NP_176280.1|](http://www.ncbi.nlm.nih.gov/entrez/query.fcgi?cmd=Retrieve&db=Protein&list_uids=15219818&dopt=GenPept&RID=SK69G8C6014&log$=protalign&blast_rank=1)ACLA-2 (ATP-citrate lyase A-2) [Arabidopsis thaliana] |
| Rs-Ra065 | AB447958 | [AT5G20950](http://www.ncbi.nlm.nih.gov/sites/entrez?db=gene&cmd=search&term=832220&RID=64K2711W01N&log$=geneexplicitprot&blast_rank=1) | 510 | 2.00E-84 | [ref|NP_197595.2|](http://www.ncbi.nlm.nih.gov/entrez/query.fcgi?cmd=Retrieve&db=Protein&list_uids=22326920&dopt=GenPept&RID=SK6CJ3W8016&log$=protalign&blast_rank=1) Glycosyl hydrolase family 3 protein(GH3) [Arabidopsis thaliana] |
| Rs-Ra067 | AB447960 | [AT1G04410](http://www.ncbi.nlm.nih.gov/sites/entrez?db=gene&cmd=search&term=839527&RID=6W6B1ASV016&log$=geneexplicitprot&blast_rank=1) | 547 | 2.00E-70 | [ref|NP_171936.1|](http://www.ncbi.nlm.nih.gov/entrez/query.fcgi?cmd=Retrieve&db=Protein&list_uids=15219721&dopt=GenPept&RID=SK6F7SZU016&log$=protalign&blast_rank=2)Malate dehydrogenase[Arabidopsis thaliana] |
| Rs-Ra030 | AB447923 | AT5G35790 | 216 | 7.00E-25 | [dbj|BAD94743.1|](http://www.ncbi.nlm.nih.gov/entrez/query.fcgi?cmd=Retrieve&db=Protein&list_uids=62321397&dopt=GenPept&RID=SJZY9VVY01N&log$=protalign&blast_rank=1) Glucose 6 -phosphate dehydrogenase (G6PD) [Arabidopsis thaliana] |
| Rs-Ra035 | AB447928 | AT1G20840 | 463 | 3.0E-46 | [ref|NP_173508.1|](http://www.ncbi.nlm.nih.gov/entrez/query.fcgi?cmd=Retrieve&db=Protein&list_uids=15218044&dopt=GenPept&RID=SK0P16AE01N&log$=protalign&blast_rank=3)TMT1(Tonoplast monosaccharides transporter1) [Arabidopsis thaliana] |
| Rs-Ra009 | AB447902 | AT1G20950 | 577 | 9.00E-89 | [ref|NP_173519.1|](http://www.ncbi.nlm.nih.gov/entrez/query.fcgi?cmd=Retrieve&db=Protein&list_uids=15218074&dopt=GenPept&RID=SJBRRZP6016&log$=protalign&blast_rank=1)Pyrophosphate-fructose-6-phosphate1-phosphotransferase-related[Arabidopsis thaliana] |
|  |  |  |  |  |  |
|  |  |  |  |  | **Unknown/ Unclassified** |
| Rs-Ra010 | AB447903 | AY835401.1 | 484 | 5.00E-16 | [gb|AAW22619.1|](http://www.ncbi.nlm.nih.gov/entrez/query.fcgi?cmd=Retrieve&db=Protein&list_uids=56693617&dopt=GenPept&RID=SJC0ZTB6014&log$=protalign&blast_rank=1)Protein Kinase C conserved region 2 (PKC) [Brassica napus] |
| Rs-Ra013 | AB447906 | AT2G26870 | 506 | 3.00E-53 | [ref|NP_180255.1|](http://www.ncbi.nlm.nih.gov/entrez/query.fcgi?cmd=Retrieve&db=Protein&list_uids=15225806&dopt=GenPept&RID=SJCXRTP5016&log$=protalign&blast_rank=1)Phosphoesterase family protein (PPEase) [Arabidopsis thaliana] |
| Rs-Ra016 | AB447909 | AT5G58540 | 545 | 1.00E-58 | [ref|NP_200662.3|](http://www.ncbi.nlm.nih.gov/entrez/query.fcgi?cmd=Retrieve&db=Protein&list_uids=145359412&dopt=GenPept&RID=SJDKMEZH016&log$=protalign&blast_rank=1)Protein kinase family protein[Arabidopsis thaliana] |
| Rs-Ra058 | AB447951 | [AT3G18490](http://www.ncbi.nlm.nih.gov/sites/entrez?db=gene&cmd=search&term=821379&RID=6W38EB3J012&log$=geneexplicitprot&blast_rank=2) | 182 | 8.00E-19 | [ref|NP_188478.1|](http://www.ncbi.nlm.nih.gov/entrez/query.fcgi?cmd=Retrieve&db=Protein&list_uids=15229656&dopt=GenPept&RID=SK4HRZHB012&log$=protalign&blast_rank=2)aspartyl protease family protein (CDN41) [Arabidopsis thaliana] |
| RaiC | AB447975 | AAZ41811 | 261 | 2.00E-25 | [gb|AAZ41811.1|](http://www.ncbi.nlm.nih.gov/entrez/query.fcgi?cmd=Retrieve&db=Protein&list_uids=71834719&dopt=GenPept) 01P13-1 [Brassica rapa subsp. pekinensis] |
| Rs-Ra062 | AB447955 | AT1G19440 | 544 | 3.00E-91 | [ref|NP_173376.1|](http://www.ncbi.nlm.nih.gov/entrez/query.fcgi?cmd=Retrieve&db=Protein&list_uids=15223556&dopt=GenPept&RID=SK5CBMMN016&log$=protalign&blast_rank=1)KCS4 (3-ketoacyl-COA synthase 4[Arabidopsis thaliana] |
| Rs-Ra051 | AB447944 | AT2G26970 | 131 | 1.00E-13 | [ref|NP_001031426.1|](http://www.ncbi.nlm.nih.gov/entrez/query.fcgi?cmd=Retrieve&db=Protein&list_uids=79323145&dopt=GenPept&RID=SKD3C70H014&log$=protalign&blast_rank=2)Exonuclease[Arabidopsis thaliana] |
| Rs-Ra052 | AB447945 | AT1G76810 | 282 | 2.00E-36 | [ref|NP_177807.3|](http://www.ncbi.nlm.nih.gov/entrez/query.fcgi?cmd=Retrieve&db=Protein&list_uids=42563275&dopt=GenPept&RID=SK3NHX0J016&log$=protalign&blast_rank=3)translation initiation factor IF-2 like protein (eIF2) [Arabidopsis thaliana] |
| Rs-Ra053 | AB447946 | AT1G11650 | 399 | 5.00E-34 | [ref|NP_849641.1|](http://www.ncbi.nlm.nih.gov/entrez/query.fcgi?cmd=Retrieve&db=Protein&list_uids=30682335&dopt=GenPept&RID=SK3T249X01S&log$=protalign&blast_rank=2) RNA binding protein 45[Arabidopsis thaliana] |
| Rs-Ra054 | AB447947 | AT3G19130 | 556 | 1.00E-44 | [ref|NP_188544.1|](http://www.ncbi.nlm.nih.gov/entrez/query.fcgi?cmd=Retrieve&db=Protein&list_uids=15230291&dopt=GenPept&RID=SK3X651301N&log$=protalign&blast_rank=2)Nuclear acid binding protein[Arabidopsis thaliana] |
| Rs-Ra055 | AB447948 | AT4G30800 | 295 | 2.00E-23 | [ref|NP_194809.1|](http://www.ncbi.nlm.nih.gov/entrez/query.fcgi?cmd=Retrieve&db=Protein&list_uids=15234873&dopt=GenPept&RID=SK414XW3014&log$=protalign&blast_rank=3)40S ribosomal protein S 11[Arabidopsis thaliana] |
| Rs-Ra056 | AB447949 | AT2G20060 | 517 | 4.00E-17 | [ref|NP_565463.1|](http://www.ncbi.nlm.nih.gov/entrez/query.fcgi?cmd=Retrieve&db=Protein&list_uids=18399235&dopt=GenPept&RID=SK45TPUR012&log$=protalign&blast_rank=2)50s ribosomal protein L4 family protein [Arabidopsis thaliana] |
| Rs-Ra050 | AB447943 | [AT5G19510](http://www.ncbi.nlm.nih.gov/entrez/utils/fref.fcgi?http://arabidopsis.org/servlets/TairObject?type=locus&name=AT5G19510) | 450 | 2.00E-68 | [ref|NP_568375.2|](http://www.ncbi.nlm.nih.gov/entrez/query.fcgi?cmd=Retrieve&db=Protein&list_uids=30687350&dopt=GenPept&RID=SKCHS20P012&log$=protalign&blast_rank=1) elongation factor 1B alpha-subunit 2 (eEF1Balpha2) [Arabidopsis  thaliana] |
| Rs-Ra046 | AB447939 | AT1G60780 | 317 | 7.00E-44 | [ref|NP_176277.1|](http://www.ncbi.nlm.nih.gov/entrez/query.fcgi?cmd=Retrieve&db=Protein&list_uids=15219810&dopt=GenPept&RID=SK2CD3K6014&log$=protalign&blast_rank=1)Clathrin adaptor complexs medium subunit family protein (Clathrin) [Arabidopsis thaliana] |
| Rs-Ra047 | AB447940 | AT2G21380 | 391 | 2.00E-38 | [ref|NP_565510.1|](http://www.ncbi.nlm.nih.gov/entrez/query.fcgi?cmd=Retrieve&db=Protein&list_uids=18399675&dopt=GenPept&RID=SK2FXZUK016&log$=protalign&blast_rank=1) kinesin motor-protin-related (Kinesin) [Arabidopsis thaliana] |
| Rs-Ra049 | AB447942 | AT3G08580 | 490 | 2.0E-71 | [ref|NP_187470.1|](http://www.ncbi.nlm.nih.gov/entrez/query.fcgi?cmd=Retrieve&db=Protein&list_uids=15231937&dopt=GenPept&RID=SK2R80HP014&log$=protalign&blast_rank=1) AAC1 (ADP/ATP CARRIER 1) [Arabidopsis thaliana] |
| Rs-Ra066 | AB447959 | AT4G34700 | 102 | 2.00E-13 | [ref|NP_567970.1|](http://www.ncbi.nlm.nih.gov/entrez/query.fcgi?cmd=Retrieve&db=Protein&list_uids=18418498&dopt=GenPept&RID=SKCZ366701N&log$=protalign&blast_rank=4) LVR family protein[Arabidopsis thaliana] |
| Rs-Ra077 | AB447969 | AT2G19760 | 385 | 1.00E-23 | [ref|NP_179566.1|](http://www.ncbi.nlm.nih.gov/entrez/query.fcgi?cmd=Retrieve&db=Protein&list_uids=15224838&dopt=GenPept&RID=SK7RY378012&log$=protalign&blast_rank=1)Profilin1 [Arabidopsis thaliana] |
| Rs-Ra080 | AB447973 | AT1G04960 | 624 | 2.00E-113 | [ref|NP_849388.1|](http://www.ncbi.nlm.nih.gov/entrez/query.fcgi?cmd=Retrieve&db=Protein&list_uids=30683070&dopt=GenPept&RID=SK6ZMBE8014&log$=protalign&blast_rank=8) Tubulin alpha-5 chain-like protein[Arabidopsis thaliana] |
| Rs-Ra081 | AB447974 | AT4G14820 | 366 | 5.00E-55 | [ref|NP_171974.1|](http://www.ncbi.nlm.nih.gov/entrez/query.fcgi?cmd=Retrieve&db=Protein&list_uids=15220329&dopt=GenPept&RID=SKAJDVKR01N&log$=protalign&blast_rank=10)similar to tubulin alpha-2[Arabidopsis thaliana] |
| Rs-Ra088 | AB447970 | AT4G29350 | 456 | 2.00E.-20 | [ref|NP_194664.1|](http://www.ncbi.nlm.nih.gov/entrez/query.fcgi?cmd=Retrieve&db=Protein&list_uids=15233538&dopt=GenPept&RID=SK80273U012&log$=protalign&blast_rank=2)PFN2 (Profilin 2) [Arabidopsis thaliana] |
| Rs-Ra063 | AB447956 | AT5G04420 | 518 | 1.00E-78 | [ref|NP_196062.1|](http://www.ncbi.nlm.nih.gov/entrez/query.fcgi?cmd=Retrieve&db=Protein&list_uids=15237715&dopt=GenPept&RID=SK65SS5901N&log$=protalign&blast_rank=2)Kelch repeat containing protein[Arabidopsis thaliana] |
| Rs-Ra083 | AB447971 | AT1G70370 | 160 | 9.00E-20 | [ref|NP_177194.1|](http://www.ncbi.nlm.nih.gov/entrez/query.fcgi?cmd=Retrieve&db=Protein&list_uids=15223133&dopt=GenPept&RID=SK9S22NJ014&log$=protalign&blast_rank=3) Polygalacturonase isoenzyme 1 beta subunit homolog (PGβ1) [Arabidopsis thaliana] |
| Rs-Ra069 | AB447962 | [AT5G47830](http://www.ncbi.nlm.nih.gov/sites/entrez?db=gene&cmd=search&term=834834&RID=6P2KC39M01R&log$=geneexplicitprot&blast_rank=1) | 241 | 3.00E-30 | [ref|NP_974902.1|](http://www.ncbi.nlm.nih.gov/entrez/query.fcgi?cmd=Retrieve&db=Protein&list_uids=42573612&dopt=GenPept&RID=SK6MZWM201N&log$=protalign&blast_rank=1)Unknown protein[Arabidopsis thaliana] |
| Rs-Ra070 | AB447963 | [AT1G33490](http://www.ncbi.nlm.nih.gov/sites/entrez?db=gene&cmd=search&term=840243&RID=6KYDMWEM016&log$=geneexplicitprot&blast_rank=1) | 254 | 3.00E-06 | [ref|NP_564425.1|](http://www.ncbi.nlm.nih.gov/entrez/query.fcgi?cmd=Retrieve&db=Protein&list_uids=18398872&dopt=GenPept&RID=SK6RGUW9016&log$=protalign&blast_rank=1)Unknown protein[Arabidopsis thaliana] |
| Rs-Ra071 | AB447964 | [AT4G13160](http://www.ncbi.nlm.nih.gov/sites/entrez?db=gene&cmd=search&term=826930&RID=6KZEWD2901R&log$=geneexplicitprot&blast_rank=1) | 662 | 1.00E-72 | [ref|NP_193052.1|](http://www.ncbi.nlm.nih.gov/entrez/query.fcgi?cmd=Retrieve&db=Protein&list_uids=15235613&dopt=GenPept&RID=SK6X4KCR016&log$=protalign&blast_rank=1)Unknown protein [Arabidopsis thaliana] |
| Rs-Ra073 | AB447966 | At5g13030 | 336 | 8.00E-51 | [ref|NP_196807.2|](http://www.ncbi.nlm.nih.gov/entrez/query.fcgi?cmd=Retrieve&db=Protein&list_uids=30684227&dopt=GenPept&RID=SK79UDKY014&log$=protalign&blast_rank=3)Unknown protein [Arabidopsis thaliana] |
| Rs-Ra074 | AB447967 | [AT3G52240](http://www.ncbi.nlm.nih.gov/sites/entrez?db=gene&cmd=search&term=824389&RID=6NRM80GS014&log$=geneexplicitprot&blast_rank=2) | 307 | 4.00E-10 | [ref|NP_190792.2|](http://www.ncbi.nlm.nih.gov/entrez/query.fcgi?cmd=Retrieve&db=Protein&list_uids=30693612&dopt=GenPept&RID=SK7EHNU5014&log$=protalign&blast_rank=2)Unknown protein [Arabidopsis thaliana] |
| Rs-Ra085 | AB447978 | XP_001234693 | 234 | 2.00E-05 | [ref|XP_001234693.1|](http://www.ncbi.nlm.nih.gov/entrez/query.fcgi?cmd=Retrieve&db=Protein&list_uids=118092801&dopt=GenPept&RID=SKBYBK8901N&log$=protalign&blast_rank=1)similar to ankyrin repeat domain 22 [Gallus gallus] |
| Rs-Ra086 | AB447979 |  | 284 |  | No significant similarity found |
| Rs-Ra087 | AB447980 |  | 334 |  | No significant similarity found |
