# Supplementary material for: Comparative transcriptional profiling-based identification of raphanusanin-inducible genes
Source: BMC Plant Biol. 2010 Jun 16;10:111. doi: 10.1186/1471-2229-10-111 (PMC3095276; doi:10.1186/1471-2229-10-111)
Supplement: Additional file 3 — Table S2: Biochemical characterization of gene clusters. [file 1471-2229-10-111-S3.DOC]

**Additional file 3**

**Table S2**

Table S2: Biochemical characterization of gene clusters

| **Clone ID** | **Accession No.** | **Locus** | **Description and putative function** |
| --- | --- | --- | --- |
|  |  |  |  |
|  |  |  | **Kinases** |
| Rs-Ra002 | AB447895 | At5g16590 | leucine-rich repeat transmembrane protein kinase (LRT) |
| Rs-Ra003 | AB447896 | [AT1G56145](http://www.ncbi.nlm.nih.gov/sites/entrez?db=gene&cmd=search&term=842067&RID=4YYBENM3012&log$=geneexplicitprot&blast_rank=3) | similar to serine/threorine kinase/leucine-rich repeat family protein |
| Rs-Ra004 | AB447897 | [AT2G30740](http://www.ncbi.nlm.nih.gov/sites/entrez?db=gene&cmd=search&term=817625&RID=6T075R6N01R&log$=geneexplicitprot&blast_rank=1) | PTI1-like protein tyrosine kinase ( Pti1 ) |
| Rs-Ra006 | AB447899 | AT2G30360 | SNF 1-related protein kinase (CIPKI) |
| Rs-Ra010 | AB447903 | AY835401.1 | Protein Kinase C conserved region 2 (PKC) |
| Rs-Ra011 | AB447904 | AT2G31880 | Leucine-rich repeat tranmembrane protein Kinase(LRR) |
| Rs-Ra012 | AB447905 | [AT5G45430](http://www.ncbi.nlm.nih.gov/sites/entrez?db=gene&cmd=search&term=834579&RID=7RZ56STW014&log$=geneexplicitprot&blast_rank=2) | serine/threonine-protein kinase (male germ cell-associated kinase) (MAK) |
| Rs-Ra016 | AB447909 | AT5G58540 | Protein kinase family protein |
| Rs-Ra038 | AB447931 | [AT5G13530](http://www.arabidopsis.org/servlets/TairObject?type=locus&name=AT5G13530) | RING E3 ligase protein (KEG) |
| Rs-Ra007 | AB447900 | [AT2G26330](http://www.ncbi.nlm.nih.gov/entrez/query.fcgi?db=gene&cmd=Retrieve&dopt=full_report&list_uids=817173) | receptor protein kinase ERECTA (ER) |
|  |  |  |  |
|  |  |  | **Protein metabolism/binding** |
| RaiA | AB-355980 | AF361759 | Cop 9 signalosome subunit 3 (CSN3) |
| Rs-Ra005 | AB447898 | AT4G03260 | Leucine-rich repeat family protein |
| Rs-Ra017 | AB447910 | AT4G24190 | SHD (SHEPHERD) ATP binding/HSP90 |
| Rs-Ra032 | AB447925 | Y11482 | Myrosinase binding protein (MBP1) |
| Rs-Ra038 | AB447931 | [AT5G13530](http://www.arabidopsis.org/servlets/TairObject?type=locus&name=AT5G13530) | RING E3 ligase protein (KEG) |
| Rs-Ra039 | AB447932 | AT4G02570 | CULLIN 1, a subunit of E3 ubiquitin ligase (CUL1) |
| Rs-Ra040 | AB447933 | [AT1G67250](http://www.ncbi.nlm.nih.gov/sites/entrez?db=gene&cmd=search&term=843045&RID=4YY38ABF012&log$=geneexplicitprot&blast_rank=1) | Proteasome maturation factor (UMP1) |
| Rs-Ra041 | AB447934 | At5g03240 | Ubiquitin (gene=UBQ3) |
| Rs-Ra042 | AB447935 | [AT3G09760](http://www.ncbi.nlm.nih.gov/sites/entrez?db=gene&cmd=search&term=820134&RID=5PTYDFRD015&log$=geneexplicitprot&blast_rank=1) | C3HC4-type Ring Finger (Zinc Finger) |
| Rs-Ra058 | AB447951 | [AT3G18490](http://www.ncbi.nlm.nih.gov/sites/entrez?db=gene&cmd=search&term=821379&RID=6W38EB3J012&log$=geneexplicitprot&blast_rank=2) | aspartyl protease family protein (CND41) |
| Rs-Ra037 | AB447930 | AT5G17920 | Cobalamin-independent methionine synthase (MetS) |
|  |  |  |  |
|  |  |  | **Singnal tranduction** |
| Rs-Ra010 | AB447903 | AY835401.1 | Protein Kinase C conserved region 2 (PKC) |
| Rs-Ra007 | AB447900 | [AT2G26330](http://www.ncbi.nlm.nih.gov/entrez/query.fcgi?db=gene&cmd=Retrieve&dopt=full_report&list_uids=817173) | receptor protein kinase ERECTA (ER) |
| Rs-Ra008 | AB447901 | AAD26119.1 | Phosphoinositide- specific phospholipase C (PLC) |
| Rs-Ra014 | AB447907 | AT3G16785 | PLDP1 (phospholipase D Zeta 1) |
| Rs-Ra001 | AB447894 | [AT5G53570](http://www.ncbi.nlm.nih.gov/sites/entrez?db=gene&cmd=search&term=835439&RID=73748WUH012&log$=geneexplicitprot&blast_rank=2) | Gtpase activator protein for Rab-like small GTPase-like protein (Gtpase) |
| Rs-Ra024 | AB447917 | [AT3G16570](http://www.ncbi.nlm.nih.gov/sites/entrez?db=gene&cmd=search&term=820907&RID=6KFM6D6R016&log$=geneexplicitprot&blast_rank=1) | RALF 23 (LILE 23) |
| Rs-Ra026 | AB447919 | [AT3G53990](http://www.ncbi.nlm.nih.gov/entrez/query.fcgi?db=gene&cmd=Retrieve&dopt=full_report&list_uids=824566) | Universal stress protein (USP) family protein |
| Rs-Ra034 | AB447927 | [AT4G23690](http://www.ncbi.nlm.nih.gov/sites/entrez?db=gene&cmd=search&term=828469&RID=61UKBTTP01R&log$=geneexplicitprot&blast_rank=4) | Disease resistence response protein (DRP) |
| Rs-Ra048 | AB447941 | [AT3G04950](http://www.ncbi.nlm.nih.gov/entrez/query.fcgi?db=gene&cmd=Retrieve&dopt=full_report&list_uids=819655) | FfG singnal recognition particle GTPase |
|  |  |  |  |
|  |  |  | **Hydrolases** |
| Rs-Ra060 | AB447953 | [AT1G17745](http://www.ncbi.nlm.nih.gov/entrez/query.fcgi?db=gene&cmd=Retrieve&dopt=full_report&list_uids=838352) | 3-Phosphoglycerate dehydrogenase (3PGD) |
| Rs-Ra013 | AB447906 | AT2G26870 | Phosphoesterase family protein (PPEase) |
| Rs-Ra022 | AB447915 | AB042187 | Myrosinase (RMB2) |
| Rs-Ra023 | AB447916 | AB042188 | Myrosinase (RMB1) |
| Rs-Ra065 | AB447958 | [AT5G20950](http://www.ncbi.nlm.nih.gov/sites/entrez?db=gene&cmd=search&term=832220&RID=64K2711W01N&log$=geneexplicitprot&blast_rank=1) | Glycosyl hydrolase family 3 protein(GH3) |
| Rs-Ra067 | AB447960 | [AT1G04410](http://www.ncbi.nlm.nih.gov/sites/entrez?db=gene&cmd=search&term=839527&RID=6W6B1ASV016&log$=geneexplicitprot&blast_rank=1) | Malate dehydrogenase |
| Rs-Ra027 | AB447920 | AT4G09750 | short-chain dehydrogenase (SDR) |
| Rs-Ra051 | AB447944 | AT2G26970 | Exonuclease |
| Rs-Ra057 | AB447950 | AT3G44260 | Ribonuclease /CCR4-NOT transcription complex protein |
| Rs-Ra030 | AB447923 | AT5G35790 | Glucose 6 -phosphate dehydrogenase (G6PD) |
|  |  |  |  |
|  |  |  | **Oxidases** |
| Rs-Ra020 | AB447913 | AF139538 | Catalase2 (CAT) |
| Rs-Ra028 | AB447921 | X81628.1 | ACC oxidase (ACCO) |
| Rs-Ra029 | AB447922 | AF375424 | Coproporphyrinogen III oxidase (CPOX) |
| Rs-Ra036 | AB447929 | AT3G32980 | Peroxidase |
| Rs-Ra033 | AB447926 | AT2G17420 | NTRA (NADPH-dependent thioredoxin reductase 2) |
|  |  |  |  |
|  |  |  | **Transcription and translation** |
| Rs-Ra018 | AB447911 | AT5G14520 | Pescadillo-related protein (Pescadillo) |
| Rs-Ra025 | AB447918 | AT2G22300 | Calmodulin-binding transcription activator 3 (CAMTA3) |
| Rs-Ra052 | AB447945 | AT1G76810 | translation initiation factor IF-2 like protein (eIF2) |
| Rs-Ra053 | AB447946 | AT1G11650 | RNA binding |
| Rs-Ra054 | AB447947 | AT3G19130 | Nuclear acid binding protein |
| Rs-Ra055 | AB447948 | AT4G30800 | 40S ribosomal protein S 11 |
| Rs-Ra056 | AB447949 | AT2G20060 | 50s ribosomal protein L4 family protein |
| Rs-Ra057 | AB447950 | AT3G44260 | CCR4-NOT transcription complex protein (CCR4-NOT) |
|  |  |  |  |
|  |  |  | **Transport and Movement** |
| Rs-Ra019 | AB447912 | AT1G19710 | Glycosyl transferase family 1 protein (GTF) |
| Rs-Ra035 | AB447928 | AT1G20840 | TMT1(Toplast Monosaccharide Transporter1) |
| Rs-Ra043 | AB447936 | AT4G19960 | potassium iron transporter (KT) |
| Rs-Ra044 | AB447937 | AT3G16180 | Proton-dependent oligopeptide transport (POT) family prtein |
| Rs-Ra045 | AB447938 | AT3G47700 | MAG2(chromosome structure maintenance protein-related) |
| Rs-Ra046 | AB447939 | AT1G60780 | Clathrin adaptor complexs medium subunit family protein |
| Rs-Ra047 | AB447940 | AT2G21380 | kinesin motor-protin-related (kinesin) |
| Rs-Ra049 | AB447942 | AT3G08580 | AAC1 (ADP/ATP CARRIER 1) |
| Rs-Ra061 | AB447954 | AT3G28710 | H+-transport two-sector ATPase |
| Rs-Ra079 | AB447972 | AT5G09870 | Cellulose synthase 5- transferase (CESA5) |
| Rs-Ra009 | AB447902 | AT1G20950 | Pyrophosphate-fructose-6-phosphate1-phosphotransferase-related (PPFP) |
| Rs-Ra062 | AB447955 | AT1G19440 | Very long-chain fatty acid condensing enzyme |
| RaiC | AB447975 | AAZ41811 | Putative Polyprotein of CRB element |
|  |  |  |  |
|  |  |  | **Energy** |
| Rs-Ra059 | AB447952 | AT5G59290 | UDP-D glucuronate carboxy-lyase |
| Rs-Ra064 | AB447957 | AT1G60810 | ACLA-2 (ATP-citrate lyase A-2) |
| Rs-Ra066 | AB447959 | AT4G34700 | LVR family protein |
|  |  |  |  |
|  |  |  | **Cellular biogenesis** |
| Rs-Ra077 | AB447970 | AT2G19760 | Profilin1 |
| Rs-Ra078 | AB447971 | AT3G18780 | Actin8/ LIGHT STRESS-REGULATED 2 |
| Rs-Ra080 | AB447973 | AT1G04960 | Tubulin alpha-5 chain-like protein |
| Rs-Ra081 | AB447974 | AT4G14820 | similar to tubulin alpha-2 |
| Rs-Ra088 | AB447970 | AT4G29350 | PFN2 (Profilin 2) [Arabidopsis thaliana] |
|  |  |  | **Unclassified** |
| Rs-Ra021 | AB447914 | AT1G14710 | Hydroxy proline rich glycoprotein family (Glycoprotein) |
| Rs-Ra031 | AB447924 | AT2G39750 | Dehydration-responsive family protein (Dehydrin) |
| Rs-Ra063 | AB447956 | AT5G04420 | Acetyl-CoA binding protein (ACBP4)/Kelch repeat containing protein |
| Rs-Ra068 | AB447961 | [AT1G13000](http://www.ncbi.nlm.nih.gov/sites/entrez?db=gene&cmd=search&term=837858&RID=6NN5VPE101R&log$=geneexplicitprot&blast_rank=1) | F3F 19.3 unknown protein |
| Rs-Ra069 | AB447962 | [AT5G47830](http://www.ncbi.nlm.nih.gov/sites/entrez?db=gene&cmd=search&term=834834&RID=6P2KC39M01R&log$=geneexplicitprot&blast_rank=1) | Unknown protein |
| Rs-Ra070 | AB447963 | [AT1G33490](http://www.ncbi.nlm.nih.gov/sites/entrez?db=gene&cmd=search&term=840243&RID=6KYDMWEM016&log$=geneexplicitprot&blast_rank=1) | Unknown protein |
| Rs-Ra071 | AB447964 | [AT4G13160](http://www.ncbi.nlm.nih.gov/sites/entrez?db=gene&cmd=search&term=826930&RID=6KZEWD2901R&log$=geneexplicitprot&blast_rank=1) | Unknown protein |
| Rs-Ra072 | AB447965 | [AT5G25265](http://www.ncbi.nlm.nih.gov/sites/entrez?db=gene&cmd=search&term=832598&RID=6PVRGW2T016&log$=geneexplicitprot&blast_rank=1) | Unknown protein |
| Rs-Ra073 | AB447966 | At5g13030 | Unknown protein |
| Rs-Ra074 | AB447967 | [AT3G52240](http://www.ncbi.nlm.nih.gov/sites/entrez?db=gene&cmd=search&term=824389&RID=6NRM80GS014&log$=geneexplicitprot&blast_rank=2) | Unknown protein |
| Rs-Ra075 | AB447968 | [AT5G22580](http://www.ncbi.nlm.nih.gov/sites/entrez?db=gene&cmd=search&term=832321&RID=65ZBMEMU016&log$=geneexplicitprot&blast_rank=1) | Unknown protein/Stress responsive A/B Barrel Domain |
| Rs-Ra083 | AB447976 | AT1G70370 | Polygalacturonase isoenzyme 1 beta subunit homolog (PGβ1) |
| Rs-Ra084 | AB447977 | AT5G15230 | GASA4 (GAST1 protein homolog) |
| Rs-Ra085 | AB447978 | XP_001234693 | similar to ankyrin repeat domain 22 |
| Rs-Ra086 | AB447979 | | No significant similarity found |
| Rs-Ra087 | AB447980 | | No significant similarity found |
