# Supplementary material for: Comparative transcriptional profiling-based identification of raphanusanin-inducible genes
Source: BMC Plant Biol. 2010 Jun 16;10:111. doi: 10.1186/1471-2229-10-111 (PMC3095276; doi:10.1186/1471-2229-10-111)
Supplement: Additional file 4 — Figure S2: RNA transcription levels of housekeeping genes tested in the raphanusanin-treated sample, presented as the CT mean value at different time points. [file 1471-2229-10-111-S4.DOC]

**Additional file 4**

**Figure S2**

Figure S2: RNA transcription levels of housekeeping genes tested in the raphanusanin-treated sample, presented as the C_T_ mean value at different time points. C_T_ values represent the mean of three replicates. The most stable and consistent control genes have the lowest slope and closest fit to a linear plot. eIf2 (third from top) had the highest and ef1α (second from top) the second highest stability indices in this experiment. Ra: raphanusanin.
