# Supplementary material for: Comparative transcriptional profiling-based identification of raphanusanin-inducible genes
Source: BMC Plant Biol. 2010 Jun 16;10:111. doi: 10.1186/1471-2229-10-111 (PMC3095276; doi:10.1186/1471-2229-10-111)
Supplement: Additional file 5 — Table S3: Summary of statistics pertaining to the stability of gene expression. [file 1471-2229-10-111-S5.DOC]

**Additional file 5**

**Table S3**

Table S3. Summary of statistics pertaining to the stability of gene expression

| **GENE** | **MEAN^a^** | **F ^b^** | **MSE ANOVA^c^** | **CV%^d^** | **SLOPE^e^** | **Stability Index^f^** |
| --- | --- | --- | --- | --- | --- | --- |
| ef1α | 23.2 | 2.9 | 1.15 | 5 | 0.1 | 0.5 |
| eIf2 | 21.9 | 1.62 | 0.83 | 3.8 | 0.19 | 0.7 |
| Ubi | 21.7 | 9.38^**^ | 3.01 | 13.9 | 0.17 | 2.4 |
| Tub | 17 | 4.66^*^ | 2.54 | 14.7 | 0.25 | 3.6 |
| Act | 17.9 | 4.3^*^ | 3.17 | 17.7 | 0.38 | 6.7 |
| L4 | 28.5 | 1.22 | 2.4 | 8.4 | 0.84 | 7 |
| 18S | 18.4 | 10.65^**^ | 3.14 | 17 | 0.55 | 9.2 |

^a^Data based on analysis of *C_T_* values. Genes are ordered, top to bottom, from those tending to show the highest stability to those showing the lowest, based on the stability of index. ^b^Approximate F-tests of variance among tissue samples tested. *, P<0.05; **P< 0.01. Degrees of freedom for the numerator were 2 and for the denominator were 15, except for 18S RNA, where these values were 2 and 6, respectively.  ^c^MSE-ANOVA represents the variance among experiments and RT-PCR reactions within experiments.; thus, MSE-ANOVA for 18S only represents within-experiment variance. ^d^Coefficient of variation (MSE divided by the mean and multiplied by 100). ^e^The slope of the regression of gene means (over experiments and samples within experiments) against overall means for the different samples. ^f^The stability index is the product of the CV and the slope (multiplication of column 4 and 5). Genes whose expression shows the least random variation within tissue samples due to variation among experiment or PCR reactions (MSE-ANOVA), and whose expression depends least (in a predictable way) on the tissue sample (slope), are preferred as controls (referred from Brunner et al. 2003) [120].
