# Supplementary material for: Comparative transcriptional profiling-based identification of raphanusanin-inducible genes
Source: BMC Plant Biol. 2010 Jun 16;10:111. doi: 10.1186/1471-2229-10-111 (PMC3095276; doi:10.1186/1471-2229-10-111)
Supplement: Additional file 6 — Materials S1. Construction of subtraction library [file 1471-2229-10-111-S6.DOC]

**Additional file 6**

**Materials S1**

**Construction of subtraction library**

Total cellular RNA was extracted using a plant RNeasy Mini kit (QIAGEN, Germany) according to the manufacturer’s instructions. This was followed by the removal of contaminating genomic DNA with an RNase-Free DNase Set (QIAGEN, Germany). To construct the subtraction library, Ra^+^-Ra^-^ (Ra^+^ stands for samples treated with raphnusanin, Ra- stands for samples without raphanusanin), Suppression subtractive hybridization (SSH) was performed using a PCR-select cDNA subtraction kit (Clontech Laboratories, U.S.A.) according to the manufacturer’s instruction. In brief, double stranded cDNA was synthesized from 2 ug of poly (A)^+^ RNA extracted from the Ra treated samples after 15 min of treatment (tester) and from the Ra un-treated samples (driver). Tester and driver cDNAs were digested with Rsal. Then, a portion of one-third of the tester cDNAs was ligated with the adaptor I:

5'-CTAATACGACTCACTATAGGGCTGAGCGGCCGCCCGGGCAGGT-3'

3'-GGCCCGTCCA-5' and another one-third was ligated with the adaptor 2R: 5'-GTAATACGACTCACTATAGGGCAGCGTGGTCGCGGCCGAGGT-3' 3'-GCCGGCTCCA-5'. In order to subtract the cDNA population present in both the tester and driver samples, each of the adaptor-ligated tester cDNAs was first hybridized in 1X hybridization buffer (Clontech) at 68°C for 12 h with excess amounts of driver cDNAs. Then, the two samples from first hybridization and freshly denatured driver cDNAs, were mixed together and hybridized in 1X hybridization buffer at 68'C overnight. Residual single-stranded cDNAs were specifically amplified by PCR with advantage cDNA polymerase mix (Clontech Laboratories, U.S.A) with a primer corresponding to the common sequence of the 5' end of the adaptors, 5'-CTAATACGCTCACTATAGGGC- 3' (primer 1). The PCR was performed 27 cycles using the following temperature profile: denaturing at 94"C for 30s, annealing primers at 66°C for 30 s, extending the primers at 72'C for 1.5 min. Next, they were amplified again with nested primers corresponding the 3' side of each adaptor, 5'-TCGAGCGGCCGCCCGGGCAGGT-3' (Nested primer 1) and 5'-AGCGTGGTCGCGGCCAGGT-3' (Nested primer 2). The PCR was performed for 12 cycles using the following temperature profile: denaturing at 94'C for 10 s, annealing primers at 68°C for 30 s, extending the primers at 72°C for 1.5 min. Finally, the subtracted and amplified cDNA were directly inserted in to the TA cloning vector, pCR® 4-TOPO vector (Invitrogen, USA).
