# Supplementary material for: Comparative transcriptional profiling-based identification of raphanusanin-inducible genes
Source: BMC Plant Biol. 2010 Jun 16;10:111. doi: 10.1186/1471-2229-10-111 (PMC3095276; doi:10.1186/1471-2229-10-111)
Supplement: Additional file 7 — Table S4: Primer sequences of seven housekeeping genes, the amplification length and the melting temperature of the amplified product. [file 1471-2229-10-111-S7.DOC]

**Additional 7**

**Table S4**

Table S4: Primer sequences of seven housekeeping genes, the amplification length and the melting temperature of the amplified product

| **Name** | **Primer sequence 5'-3'** | **Primer sequence 5'-3'** | **Length (bp)** | ***T*_m_ (°C)** |
| --- | --- | --- | --- | --- |
| ef1α | CTCAACAGCACGAACACACTC | GGTGACGAGACAGAGGAGGA | 168 | 65 |
| if2 | TCGATATACGCCTTGAACTGG | TCGACGTGAAAGTGACCACAGA | 112 | 65 |
| Ubiquitin3 | CGTCTCCGTGGTGGTATGC | CCTTCCTTGTCCTGAATCTTAGCC | 119 | 65 |
| Tubulin | TGGTGGAGGAACTGGATCTGG | GGTGGAGACCTGTGGAGATGG | 115 | 65 |
| Actin8 | GCTCCTGCTATGTATGTCG | CACACCATCACCAGAGTCG | 94 | 65 |
| L4 | CGATAGGACCGCCTTCTACA | TATGCAGCTCCTCGTGTTTG | 122 | 65 |
| 18s rRNA | GGAGAGGGAGCCTGAGAAAC | CCTCCAATGGATCCTCGTTA | 168 | 65 |
