# Supplementary material for: Comparative transcriptional profiling-based identification of raphanusanin-inducible genes
Source: BMC Plant Biol. 2010 Jun 16;10:111. doi: 10.1186/1471-2229-10-111 (PMC3095276; doi:10.1186/1471-2229-10-111)
Supplement: Additional file 8 — Table S5: Primer sequences of fifty ESTs, the amplification length and the melting temperature of the amplified product. [file 1471-2229-10-111-S8.DOC]

**Additional file 8**

**Table S5**

Table S5: Primer sequences of fifty ESTs, the amplification length and the melting temperature of the amplified product

| **Family** | **Primer sequence 5'-3'** | **Primer sequence 5'-3'** | | **Length (bp)** | ***T*_m_ (°C)** |
| --- | --- | --- | --- | --- | --- |
| RALF | GGTACCATAGCCGAATGCTC | GGGCAGGTACCTCCTGTTC | | 141 | 65 |
| G6PD | TAGCAAGGGAGGAAAGGCTTA | GTACAAGTTTCCAGGGACGTG | | 208 | 65 |
| MBP1 | CGGGTCCTTCCTTTGTCTGT | TGAATGGGATGATGGAGGTG | | 119 | 65 |
| Kinesin | GCTTTTCTCCCTGGCCTTGT | CAAGAGACTTAGCGGCAGCA | | 91 | 65 |
| ACCO | AGCCGTCCGGTATTCATCAG | AGGATGTCGATTGGGAAAGC | | 95 | 65 |
| ERECTA | GCCGTGAAGAAGGTCCCATA | TCCCACAACAACCCACAGTC | | 171 | 65 |
| PPFP | GGGTATGATTTGCTGGGAAG | GAGCGGCATCTGTGTTTGAT | | 139 | 65 |
| CIPKI | GTCGCTGTTGGCGTCTGAT | ATTTGCTCGTGGTGGTGGA | | 121 | 65 |
| CUl1 | TGGTCTATGTCGTGGAGTGC | TGAGGATGTTATCGCAGAATG | | 141 | 65 |
| NTRA | TCCTCCATAGCCGAACCTC-65 | TCCCGCTCTCATCTCCATAC | | 150 | 65 |
| RMB1 | TGAAACACACTGCCCAGGAT | GTTCTCGGTGGCGTAGATTA | | 190 | 65 |
| RMB2 | CCAATCGCATAGCCTCTTGT | CTGGGACCTTCCTCAAACAC | | 177 | 65 |
| GTPase | TATAATAGCATGGCGGGTCAG | TTTGGCTTTCATTTCACTTCA | | 145 | 65 |
| LRT | CTCTCGTCTGGTCCGTCTTC | TTCTGGGATCTTGCCTGAGA | | 136 | 65 |
| GH3 | GCGTCATCAATCCTGCTCA | ATCACAAACCCTCCGCATC | | 161 | 65 |
| BURP | CCCGGTTCAGAGACATTCAC | GCCGTTTTTGGCGTAGTCTT | | 108 | 65 |
| GASA4 | CGATTGGGATGTTTTCAAGG | TGTGCCTCCGGGTTACTATG | | 102 | 65 |
| CAT | TGGAAGGGTTCGGTGTCC | GGCTGTGGTTGGCTCCTC | | 144 | 65 |
| 3PGD | CTGTCTCTGATGCTGGTGCT | GTTCTGCTCCCCGAGTATGT | | 179 | 65 |
| PKC | GAGCAAAGCAGGGAGGTTCA | TCGGTCTCGCTATTCACAGG | | 90 | 65 |
| Dehydrin | TGTTTGGTTCCGTCTCCTAAA | AACTTGCTCTTGTCTCGTGGA | 155 | | 65 |
| HSP90 | TGTCAAAAGAAGGACTGAAGG | CGTCACAACTACACAGGGAGTG | | 164 | 65 |
| Pescadillo | CGGCTCATAACACCAGCGTA | GTGGACAACGAAGCAGAAGG | | 155 | 65 |
| CAMTA3 | GAGAACGATGACGGGTTGG | TCGGACCTTTTCCACCTTC | | 142 | 65 |
| MAK | AGATTTTCTGGCTTTAGATCACG | GGATCGTCCTCAGCTTTTTG | | 120 | 65 |
| USP | TCTTCCTTGAACCAGTGTCG | AATCTCGCAACGCCCTCT | | 141 | 65 |
| PMF | CTGGAAAGAGATCGGACGAG | AGCATTGACGAAGGAATTGG | | 142 | 65 |
| SDR | GAGACGAGCTTCTCCTTAGGC | GGCCGAGGTACAAGAACAAAG | | 184 | 65 |
| CND41 | ATGATGAGGTGTCGTCGTTG | GAGTCGCCGGAATCTTCAC | | 93 | 65 |
| PLC2 | GAGTGTTGTTTTTACAGGCAGAG | CAAGGGATGTTTAGAGCCAAT | | 123 | 65 |
| CCR4-NOT | AATGTCACCAAGCCGGTTC | GCCGAGGTACAAAACACCAG | | 116 | 65 |
| CPOX | ATGGAGTTTACAGAGCAGCACA | TGACAGTGGAAGAGAGACAAA | | 147 | 65 |
| Peroxidase | CGATTTCAACCACATCTTGGA | CCACTGACACACTCCCCTTG | | 183 | 65 |
| MetE | CAGCCCAGTCCAAGTAGAAAG | TGGTCCTTCGTCAGAAACG | | 184 | 65 |
| MAG2 | ATCTTCTTGGCTTCGGCTTC | GGACATGGAGGTTCTGTATGG | | 192 | 65 |
| DRP | GCTGACGTTGCGTTTGCTAC | CCCGGAAAACAATGGATCAG | | 100 | 65 |
| POT | TATCGCGGTTCTCCTTCTGT | CATCCTCCCATTAGCTTCCA | | 142 | 65 |
| Zinc Finger | GAAGAAAACCGAGTCCTTGC | GCTGGAACGGAGCGTGAT | | 141 | 65 |
| Clathrin | TCTTGGATGTGATTGAGAATGTG | TTTTCCCTTTGTTGCTCGTC | | 176 | 65 |
| 3KCS4 | TCCACCAGCATGGATACAGA | ACGTTGGGTCCTCTTGTGTT | | 147 | 65 |
| CESA5 | ACAAAGTCCACCCTGCATTC | CCGTCTTGCATAGTCCAACC | | 133 | 65 |
| GTF | CGCAGGTAAAGACGGTGTGTC | CCTTTCTTCCCCATTGTCGTC | | 90 | 65 |
| CSN3 | TGGCCAGTTTAGTGGCAGTCT | GTAAAGGGATGCCACTGCTTG | | 207 | 65 |
| PPEase | TGCTTGGATGGATGAAGAAAC | AATGTGAACCTGACCCGAAC | | 120 | 65 |
| HPR | GGTGTTTTCCTCCCACCAG | CGGCTTCTTCTTTCGTCTTC | | 118 | 65 |
| PTI1 | CGAAGACTACCAAGCGAAGG | CAAGAAGCACAACCCCAAAG | | 179 | 65 |
| KT | CCAAGGAGTTCCTTCGTCAG | GGCTCTGATTCTTGAAACTAGCA | | 90 | 65 |
| KEG | GTGGGTGTTGATGCTAATGG | CTAGAACGATGTCAGCAGGAT | | 91 | 65 |
| Profilin | TTGCATTGACCACCAGTCAT | TTATTCAAGGTGAGCCAGGAG | | 127 | 65 |
| UBQ3 | CGTCTCCGTGGTGGTATGC | CCTTCCTTGTCCTGAATCTTAGCC | | 119 | 65 |
